# Multi-platform omics analysis of Nipah virus infection reveals viral glycoprotein modulation of mitochondria

**DOI:** 10.1016/j.celrep.2025.115411

**Published:** 2025-03-17

**Authors:** Gunner P. Johnston, Fikret Aydemir, Haewon Byun, Emmie de Wit, Kristie L. Oxford, Jennifer E. Kyle, Jason E. McDermott, Brooke L. Deatherage Kaiser, Cameron P. Casey, Karl K. Weitz, Heather M. Olson, Kelly G. Stratton, Natalie C. Heller, Viraj Upadhye, I. Abrrey Monreal, J. Lizbeth Reyes Zamora, Lei Wu, D.H. Goodall, David W. Buchholz, Joeva J. Barrow, Katrina M. Waters, Ruth N. Collins, Heinz Feldmann, Joshua N. Adkins, Hector C. Aguilar

**Affiliations:** 1 Department of Microbiology and Immunology, College of Veterinary Medicine, Cornell University, Ithaca, NY 14853, USA; 2 Laboratory of Virology, Division of Intramural Research, National Institute of Allergy and Infectious Diseases, National Institutes of Health, Rocky Mountain Laboratories, Hamilton, MT 59840, USA; 3 Earth and Biological Sciences Directorate, Pacific Northwest National Laboratory, Richland, WA 99354, USA; 4 Department of Molecular Microbiology and Immunology, Oregon Health & Science University, Portland, OR 97239, USA; 5 National Security Directorate, Pacific Northwest National Laboratory, Richland, WA 99354, USA; 6 Division of Nutritional Sciences, College of Human Ecology, Cornell University, Ithaca, NY 14853, USA; 7 Department of Molecular Medicine, College of Veterinary Medicine, Cornell University, Ithaca, NY 14853, USA; 8 These authors contributed equally; 9 Lead contact

## Abstract

The recent global pandemic illustrates the importance of understanding the host cellular infection processes of emerging zoonotic viruses. Nipah virus (NiV) is a deadly zoonotic biosafety level 4 encephalitic and respiratory paramyxovirus. Our knowledge of the molecular cell biology of NiV infection is extremely limited. This study identified changes in cellular components during NiV infection of human cells using a multi-platform, high-throughput transcriptomics, proteomics, lipidomics, and metabolomics approach. Remarkably, validation via multi-disciplinary approaches implicated viral glycoproteins in enriching mitochondria-associated proteins despite an overall decrease in protein translation. Our approach also allowed the mapping of significant fluctuations in the metabolism of glucose, lipids, and several amino acids, suggesting periodic changes in glycolysis and a transition to fatty acid oxidation and glutamine anaplerosis to support mitochondrial ATP synthesis. Notably, these analyses provide an atlas of cellular changes during NiV infections, which is helpful in designing therapeutics against the rapidly growing *Henipavirus* genus and related viral infections.

## In brief

This study identified changes in cellular components that occur during infection of human cells by the deadly Nipah virus (NiV). We used a multi-platform, high-throughput transcriptomics, proteomics, lipidomics, and metabolomics approach, providing an atlas of cellular changes helpful in designing therapeutics against the rapidly growing and important *Henipavirus* genus.

## Graphical Abstract

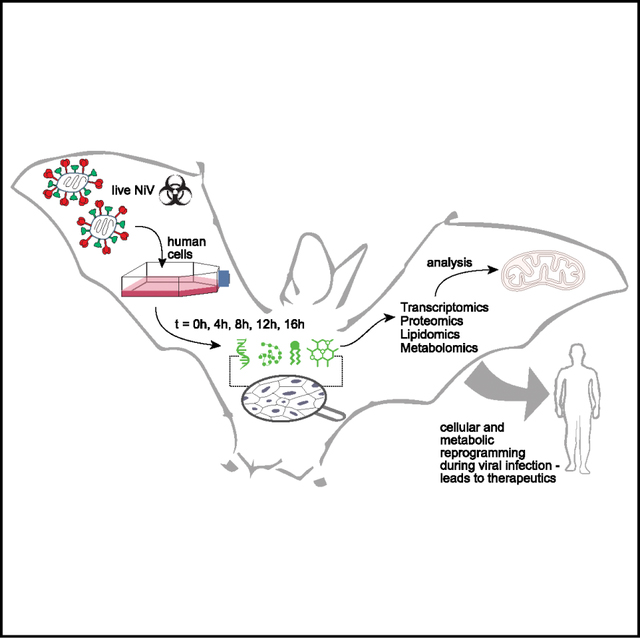

## INTRODUCTION

The current global coronavirus pandemic illustrates the importance of understanding emerging zoonotic viral infections, particularly when they result in high host mortality rates. The genus *Henipavirus* in the family Paramyxoviridae is a rapidly growing group of emerging pathogens of bat origin. It includes Nipah virus (NiV) and Hendra virus (HeV), agents requiring biosafety level 4 (BSL-4) containment. Outbreaks of NiV have occurred almost annually since its discovery two decades ago in Malaysia, with human mortality rates between 40% and 100%, including the 2018 outbreak in India, with a mortality rate of 94%.^1–5^ Within the last decade, over twenty novel henipaviral sequences have been identified across Asia, Oceania, Africa, and Central and South America, primarily in bats.^6–9^ Further, the reservoir species for these viruses have expanded to include multiple genera of bats and potentially rodents.^7,9,10^ Despite the biodefense implications and pandemic potential, approved treatments and vaccines for human use remain to be developed and approved.

The NiV negative-sense single-stranded RNA genome contains six genes in the 3′−5′ order: nucleoprotein (N), phosphoprotein (P), matrix protein (M), fusion glycoprotein (F), attachment glycoprotein (G), and large polymerase (L). Virus entry requires NiV G to bind the highly conserved host protein receptor ephrinB2 or ephrinB3, inducing a sequential conformational cascade in G and F, respectively. After entry, the viral genome is transcribed by the N, P, and L proteins,^11,12^ which work together to allow viral genome replication.^13^ Later during infection, NiV G and F on the infected cell surface drive cell-cell fusion (syncytia formation) through a similar membrane fusion process, and large, multinucleated syncytia can be observed in NiV infections in patients.^14^

The use of highly conserved host cell protein receptors enables NiV to infect a wide range of mammalian hosts, including but not limited to bats, pigs, cats, dogs, horses, rodents, and humans, making NiV prime for zoonosis.^2,9,15–21^ NiV transmission to humans has occurred from other humans, infected livestock (particularly pigs), and bats via consumption of contaminated fruit such as date palm sap.^1,4,21–24^ NiV infection in humans is largely observed in the respiratory epithelia, the central nervous system (CNS), and the microvascular endothelial tissues of major organs, including the lung, spleen, kidney, and heart.^14,17,25,26^ Animal models suggest initial infection in respiratory and olfactory epithelia,^16,18,27^ followed by the CNS via the olfactory nerves.^28^ The NiV P gene produces the alternative proteins V, W, and C, which inhibit multiple innate immune pathways, contributing to virulence.^29–32^ M, involved in virion assembly and egress, also directly inhibits immune responses, such as the interferon I response, via interaction with TRIM6.^33^

Significant advances in omics methodologies used for high-throughput analyses have allowed for an increasingly broad and complete understanding of dynamic processes, such as viral infection at a molecular level.^34–41^ These high-throughput approaches, however, have largely not been applied to BSL-4 pathogens, likely due to the technical and logistical difficulties of preparing BSL-4 samples for BSL-2 analyses while maintaining sample quality. Whereas, recently, high-throughput methods have been used to elucidate several aspects of the NiV life cycle, such as particle formation and protein interactions, application in the context of full virus infection has remained extremely limited.^42,43^ Here, we describe a multi-platform omics study of NiV infection of human embryonic kidney (HEK293T) cells, a cell line widely used for studying henipaviral infections, entry, and assembly.^42,44–48^ Specifically, we used a mass-spectrometry-compatible *irradiation* inactivation approach to allow for analyses by transcriptomics, proteomics, metabolomics, and lipidomics at multiple time points. Our findings include a general timeline for the detectable expression of each viral protein as well as major changes to host protein expression, bioinformatically sorted processes, and cellular locations. Further, lipidomics and metabolomics analyses supplemented the proteomics analysis to provide unique perspectives into the metabolic dynamics of an infected cell. The involvement of mitochondria was identified and validated by several multi-disciplinary approaches, including the transient expression of individual NiV genes, implicating NiV F and G in mitochondrial protein expression. Lastly, to our knowledge, our omics findings are the most comprehensive analysis to date for the henipaviruses or the paramyxoviruses using a multi-platform approach across multiple time points.

## RESULTS

### Experimental design of NiV infection multi-platform omics analyses

**Figure 1.**
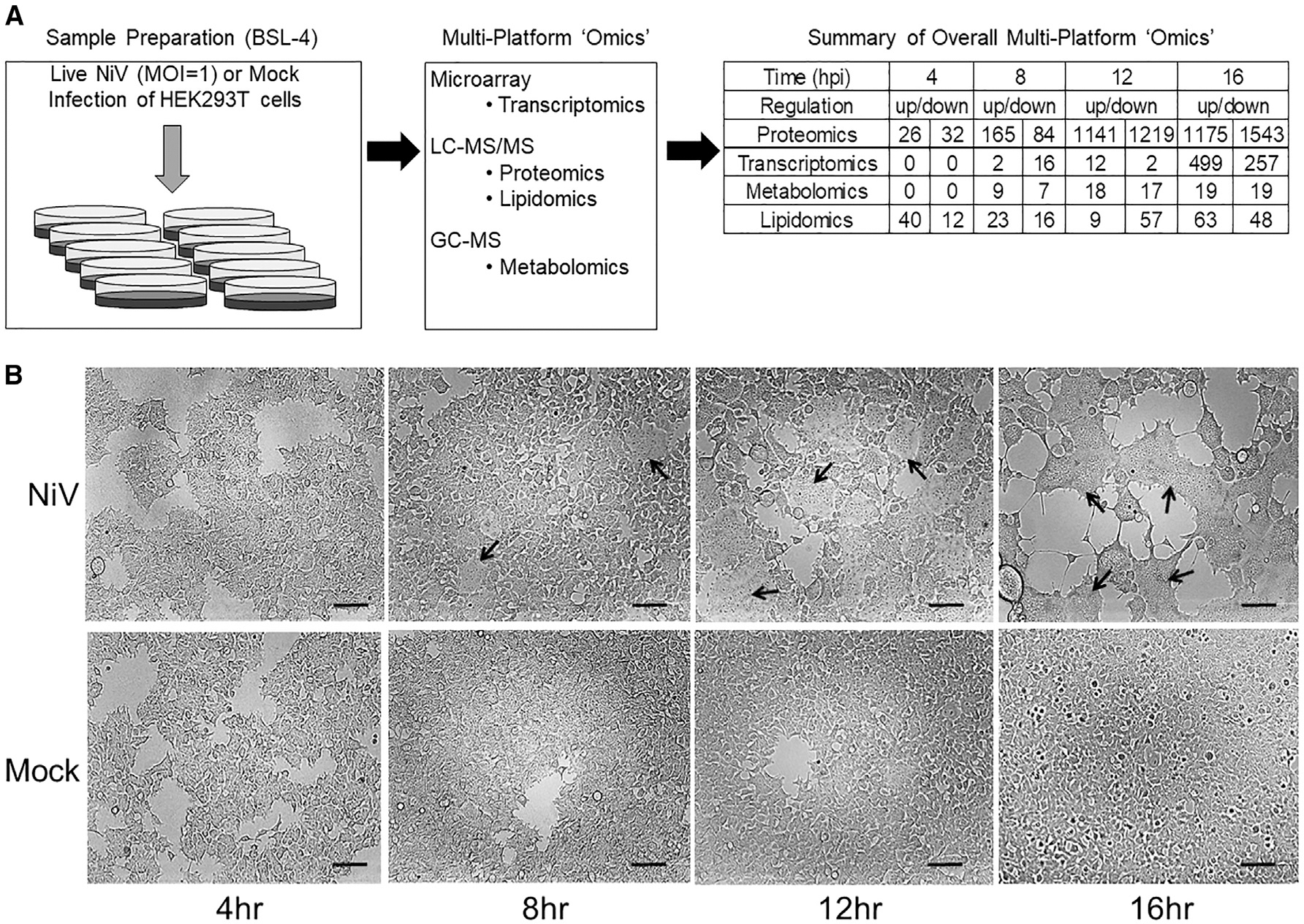
Overview of Nipah virus infection omics study (A) Five pairs of mock and NiV infections were made at a multiplicity of infection (MOI) of 1 in HEK293T cells for each of four time points (4, 8, 12, and 16 h post-infection). All the biomolecules are listed depending on their abundance. (B) Formation of cell-cell fusion (syncytial formation) (black arrows) starting at 8 hpi and increases during infection to almost complete fusion at 16 hpi. Scale bars, 100 μm.

HEK293T cells were either mock or NiV infected in five replicates at a multiplicity of infection (MOI) of 1 and collected 4, 8, 12, or 16 h post infection (hpi) (Figure 1A). We used a combination of an irradiation strategy and a recently developed preparation approach, Metabolite, Protein, and Lipid Extraction (MPLEx), to achieve the full inactivation of samples while maintaining analyte integrity to successfully assess proteomics, lipidomics, and metabolomics simultaneously.^49^ Additional cell infections were carried out for transcriptomics analysis, and the infected cells were treated with a TRizol reagent that inactivates the virus.^50–54^ A summary for the numbers of transcripts, proteins, lipids, and metabolites that met both statistical and biological thresholds (*p* < 0.05 and fold change [FC] ≤ −1.5 or ≥ 1.5) are reported as differentially expressed for each time point. For example, we report that at the 12-h time point, nearly equal numbers of cellular proteins met significance (*p* < 0.05) and FC (±1.5) thresholds for classification as increased (1,141 proteins) and decreased (1,219 proteins) in abundance compared to time-matched mock infection controls.

### Detection of NiV proteins during infection

To understand the kinetics of infection, we examined the abundance of viral and cellular proteins across time points. The abundance of paramyxoviral transcripts and proteins decreases from N to L.^55^ Although the 4 hpi time point was assessed, the first statistically significant identifications of NiV protein peptides, as compared to the time-matched mock control samples, were for N, P (or V or W), M, and G, starting at 8 hpi (Table S1). While individual changes to protein abundance at 4 hpi are likely important, the low numbers of changes made finding consistent patterns non-viable. Therefore, our analyses primarily focused on time points 8, 12, and 16 hpi. The initial identification of viral proteins largely fits the expectation for the order of viral gene expression for similar viruses.^45^ Starting at the 12-hpi time point, all NiV proteins were detected (Table S1). Additionally, 12 hpi was the first time point from which new infectious virus could be collected (data not shown). Cell-cell fusion was first noted at 8 hpi (Figure 1B), with increasing levels at 12 hpi, and by 16 hpi, most cells were fused. Since all viral proteins were highly expressed between 12 and 16 hpi, the infected cells were expected to undergo the most drastic changes at these time points. In a parallel experiment, more extensive cell-cell fusion and syncytia floating in the supernatant were observed at 24 hpi.

### Proteomic analyses identified highly significant increases to proteins associated with RNA processing and mitochondria

**Figure 2.**
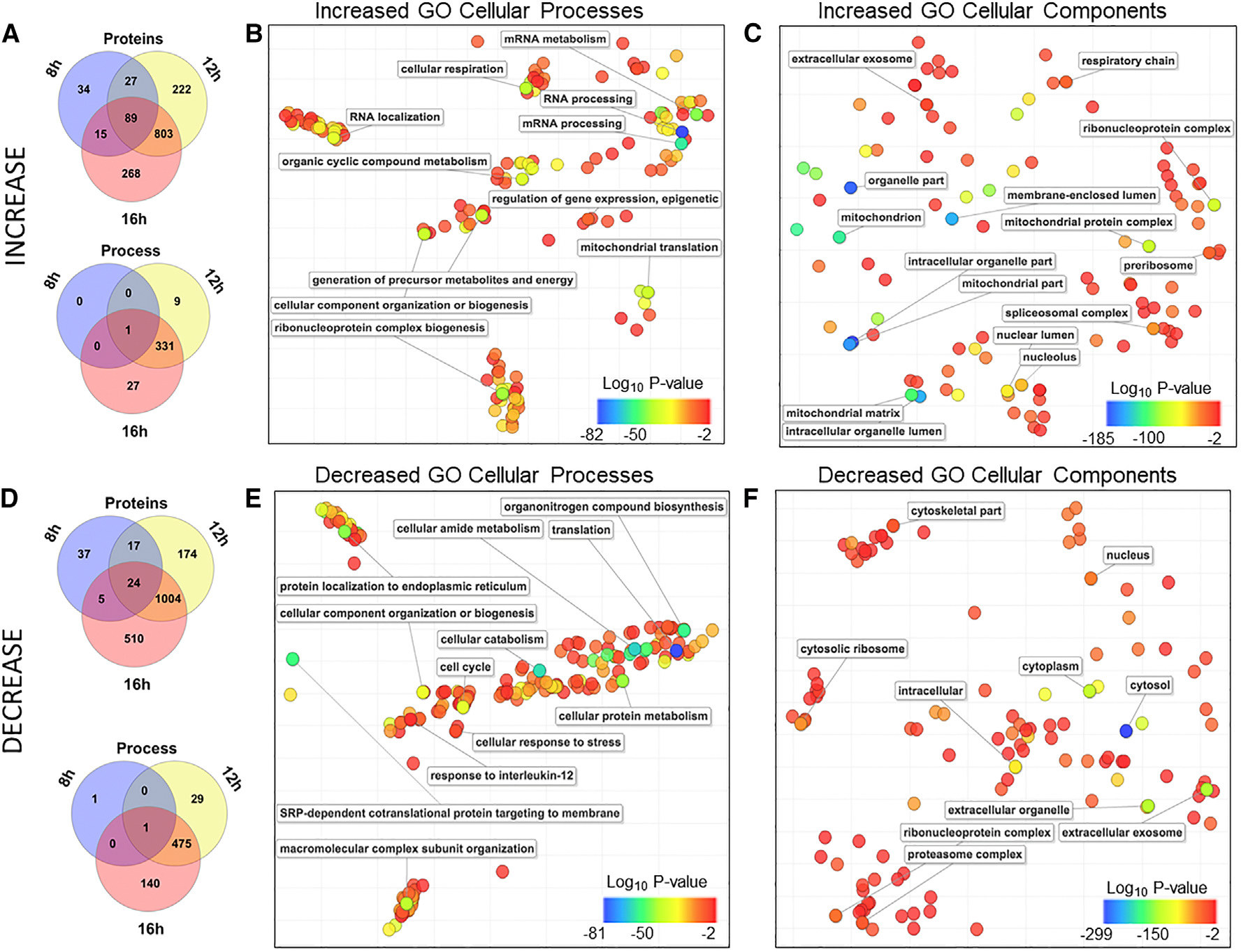
Proteomic analyses identified highly significant increases to proteins associated with RNA processing and mitochondria (A) Significantly increased (*p* < 0.05; log_2_FC ≥ 0.58) proteins were presented using InteractiVenn. (B) Increased cellular processes are mapped using GOTERMFINDER. (C) Increased subcellular components and locations are mapped using REVIGO. (D) Significantly decreased (*p* < 0.05; log_2_FC ≤ −0.58) proteins were presented using InteractiVenn. (E) Decreased cellular processes are mapped using GOTERMFINDER. (F) Decreased subcellular components and locations are mapped using REVIGO.

Comparison of proteins of increased abundance during infection demonstrated a high degree of similarity between changes at 12 and 16 hpi, not only in terms of individual proteins (Figure 2A, top) but also for Gene Ontology (GO) enrichment categories of cellular processes (Figure 2A, bottom). To reduce the high levels of redundancy between the plethora of GO terms enriched from our protein lists, we used another software, REVIGO, that consolidates similar terms.^56^ REVIGO also makes use of multidimensional scaling plots, which help describe complicated data normally requiring more than three dimensions, to spatially cluster similar GO terms in two dimensions. Based on the GO term overlap between 16 hpi and the other time points, only 16-hpi plots for processes and cellular localization were shown for simplicity (Figures 2B and 2C). In terms of protein abundance, the most *up-regulated* cellular processes (Figure 2B) included RNA processing (*p* = 10^−82^), ribonucleoprotein complex biogenesis (*p* = 10^−44^), cellular respiration (*p* = 10^−42^), generation of precursor metabolites and energy (*p* = 10^−39^), and mitochondrial translation (*p* = 10^−39^). Increased proteins were most associated with mitochondrial compartments (*p* = 10^−162^), ribonucleoprotein complexes (*p* = 10^−83^), and nuclear lumen (*p* = 10^−59^), nucleoli (*p* = 10^−48^), and spliceosomal (*p* = 10^−39^) complexes (Figure 2C).

### Proteins associated with the cytosol, exosomes, and translation were most likely to be reduced during infection

Using similar analyses, we assessed significantly *decreased* protein abundance profiles over the course of NiV infection (Figure 2D). As with proteins of *increased* abundance, the lists of proteins and GO terms identified at 8 and 12 hpi overlapped substantially with and were exceeded in number by lists for the 16-hpi time point (Figure 2D). The most consistently down-regulated cellular processes (Figure 2E) included translation (*p* = 10^−81^), cellular catabolism (*p* = 10^−61^), signal recognition particle (SRP)-dependent co-translational protein targeting to membrane (*p* = 10^−56^), and cell cycle (*p* = 10^−35^). In contrast to major abundance enrichment for mitochondria proteins, reductions in the abundance of proteins were most associated with the cytosol (*p* = 10^−299^) and extracellular exosomes (*p* = 10^−154^) (Figure 2F). Additional groups with significant reductions in abundance included cytosolic ribosomes (*p* = 10^−60^), nuclei (*p* = 10^−51^), proteasome complexes (*p* = 10^−30^), and the cytoskeleton (*p* = 10^−30^).

### Reactome mapping of cellular proteomic changes 16 h after NiV infection

**Figure 3.**
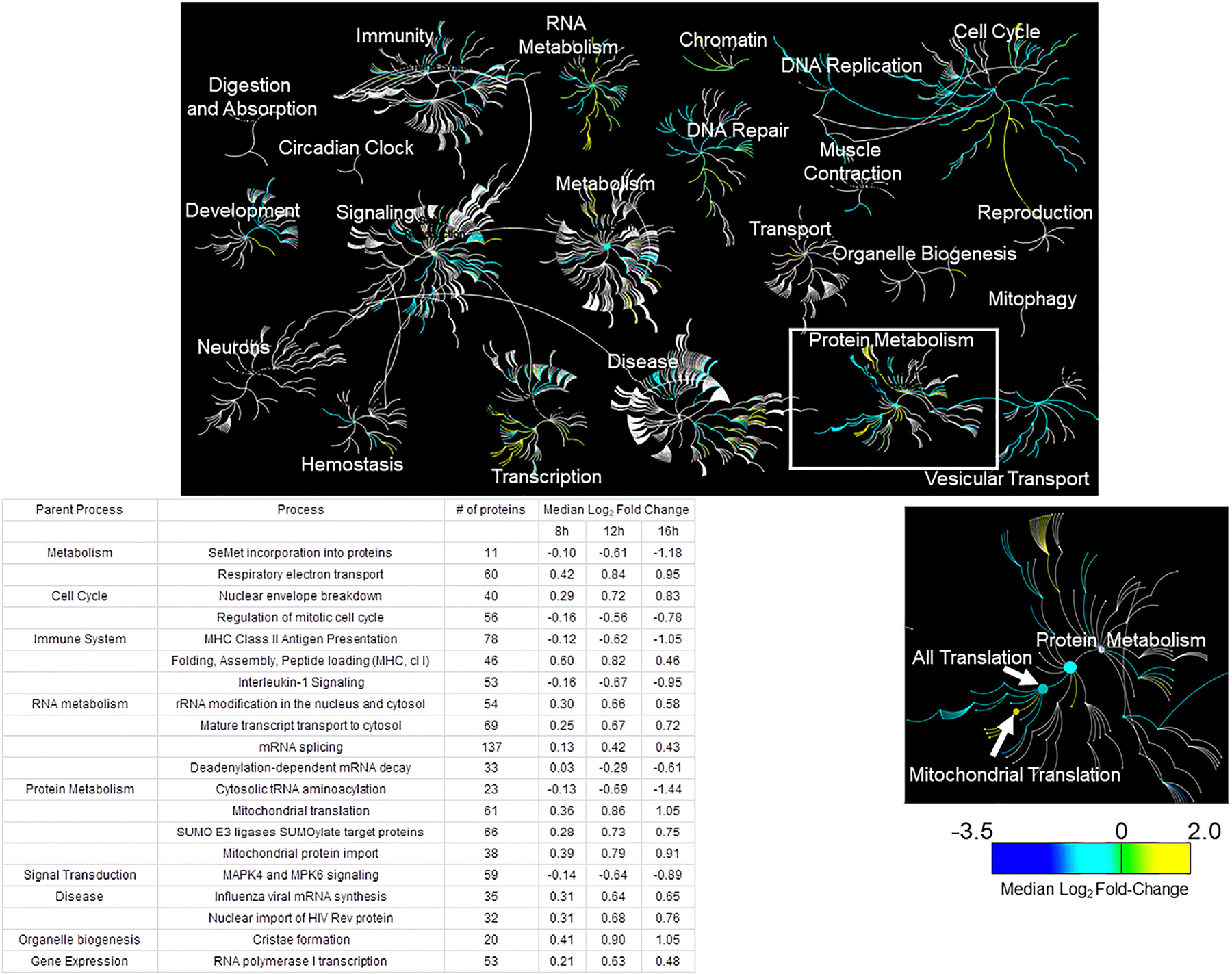
Proteins associated with the cytosol, exosomes, and translation were most likely to be reduced during infection Statistically significant proteins are grouped in main cellular pathways (nodes). Branches display where individual proteins are integrated. The heatmap displays the relative abundance.

To supplement our GO analyses, which identified processes and cellular locations most *statistically* affected by infection, we utilized another analysis approach focused on summarizing average *FCs* for proteins within the cellular pathways most affected. For this purpose, the pathway analysis tool associated with the Reactome database was used.^57^ Specifically, all proteins identified as passing both statistical (*p* ≤ 0.05) and biological (FC ≥ 1.5 or ≤ −1.5) thresholds for at least one time-point were compiled along with their associated FCs for each time point. This list is included in the supplemental information (Table S2) to support further reader analyses of these data in Reactome. An overview of many pathways found to be affected at 16 hpi is shown in Figure 3. This pathway overview organizes many cellular functions/pathways into several clusters of branches. Each of these branched groups has a central node, which designates the most general pathway in the center (Figure 3, inset shows the example “protein metabolism”). Related pathways that can be categorized under this central node are designated by nodes of increasing specificity further from the central node. This is clear in the case of the protein metabolism cluster (Figure 3, inset), where one of the next most general pathways is “all translation.” Increasing specificity once more, one of the sub-pathways under all translation is “mitochondrial translation.” This pattern is repeated, creating the branching appearance extending from each central node/process.

The Reactome pathway map (Figure 3) indicates which cellular pathways are significantly affected through the inclusion of color. Specifically, the color of each node (i.e., pathway) corresponds to the *average* FC (FCs were log_2_ transformed for normalization) in the abundance of proteins belonging to that pathway. Thus, the pathway map in Figure 3 demonstrates that at 16 hpi, NiV-infection-associated proteomic changes impact many diverse cellular pathways. One of the most interesting findings from this pathway map was that proteins involved in translation generally exhibited substantial decreases in abundance except in the case of mitochondrial translation machinery, which was clearly increased (Figure 3, inset). This finding might help explain why at least some mitochondrial proteins, particularly those made within these organelles, exhibited increased abundance compared to mock infections. The consistent and substantial increases in mitochondrial proteins indicate that their function has important roles in supporting viral spread, potentially for the provision of contact sites at the subcellular complexes for viral events.

### Transcriptional but not proteomic changes indicate protein folding stress response during late infection

**Figure 4.**
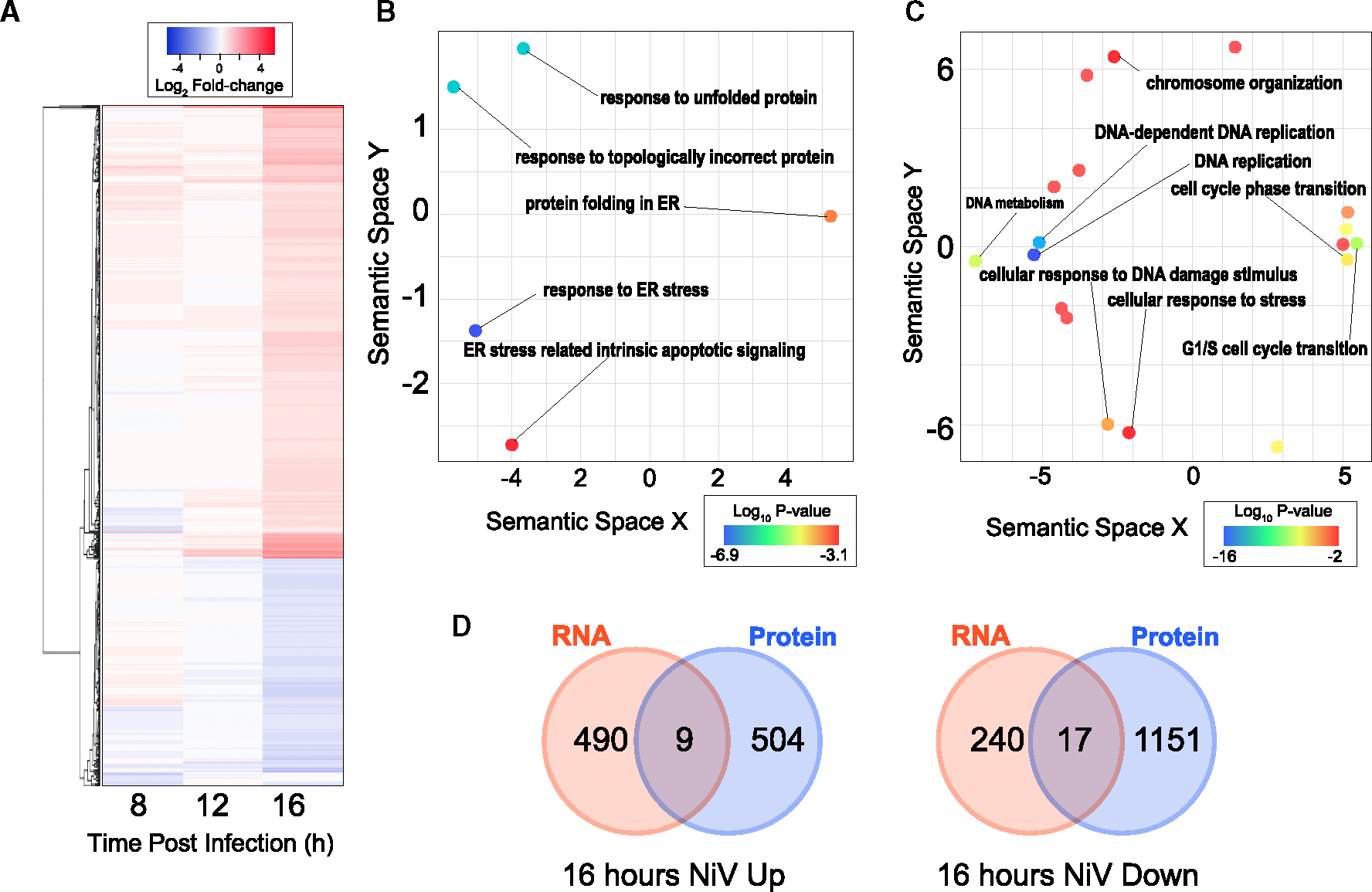
Transcriptional changes during late infection (A) The microarray data are summarized in a heatmap demonstrating visual representation of transcriptional modulation during infection. (B) REVIGO is used for process enrichment, up-regulation. Log_10_ Bonferroni-corrected *p* values were calculated for each GO term enrichment. (C) REVIGO is used for process enrichment, down-regulation. Log_10_ Bonferroni-corrected *p* values were calculated for each GO term enrichment. (D) Limited overlap between transcriptomics and proteomics is highlighted in Venn diagrams.

Transcriptomics analyses (Figure 4A), showing visual representations of the amount changed, also indicated increasingly dramatic changes in cellular processes from 8 to 12 to 16 hpi (Table S7). Changes were clearest at the 16-hpi time point, indicating increased expression of transcripts associated with the endoplasmic reticulum (ER) stress response (*p* = 10^−7^) (Figure 4B) as well as reduced transcripts associated with DNA replication (*p* = 10^−16^) and the G1/S cell cycle transition (*p* = 10^−9^) (Figure 4C). Of particular interest for these pathways was the identification of protein kinase R (PKR) as increasingly reduced in abundance since PKR is centrally involved in numerous major stress and antiviral response pathways, including the activation of nuclear factor κB (NF-κB) signaling after recognition of viral double-stranded RNA (dsRNA).^58,59^ Importantly, several mechanisms for combatting the antiviral functions of PKR have been developed by viruses, including PKR targeting for degradation by some Rift Valley fever virus proteins.^60,61^ We subcategorized the genes regarding their regulation pattern; up and down. Interestingly, neither the up-regulated nor the down-regulated proteins corresponded to their respective up- or down-regulated transcripts (Figure 4D). Given that this lack of overlap was also consistent when comparing cellular processes affected during infection (Figures 2, 3, and 4), post-transcriptional regulation is very likely to play major roles in shaping the proteome.

### Metabolomics and lipidomics indicate increased glucose and lipid metabolites

**Figure 5.**
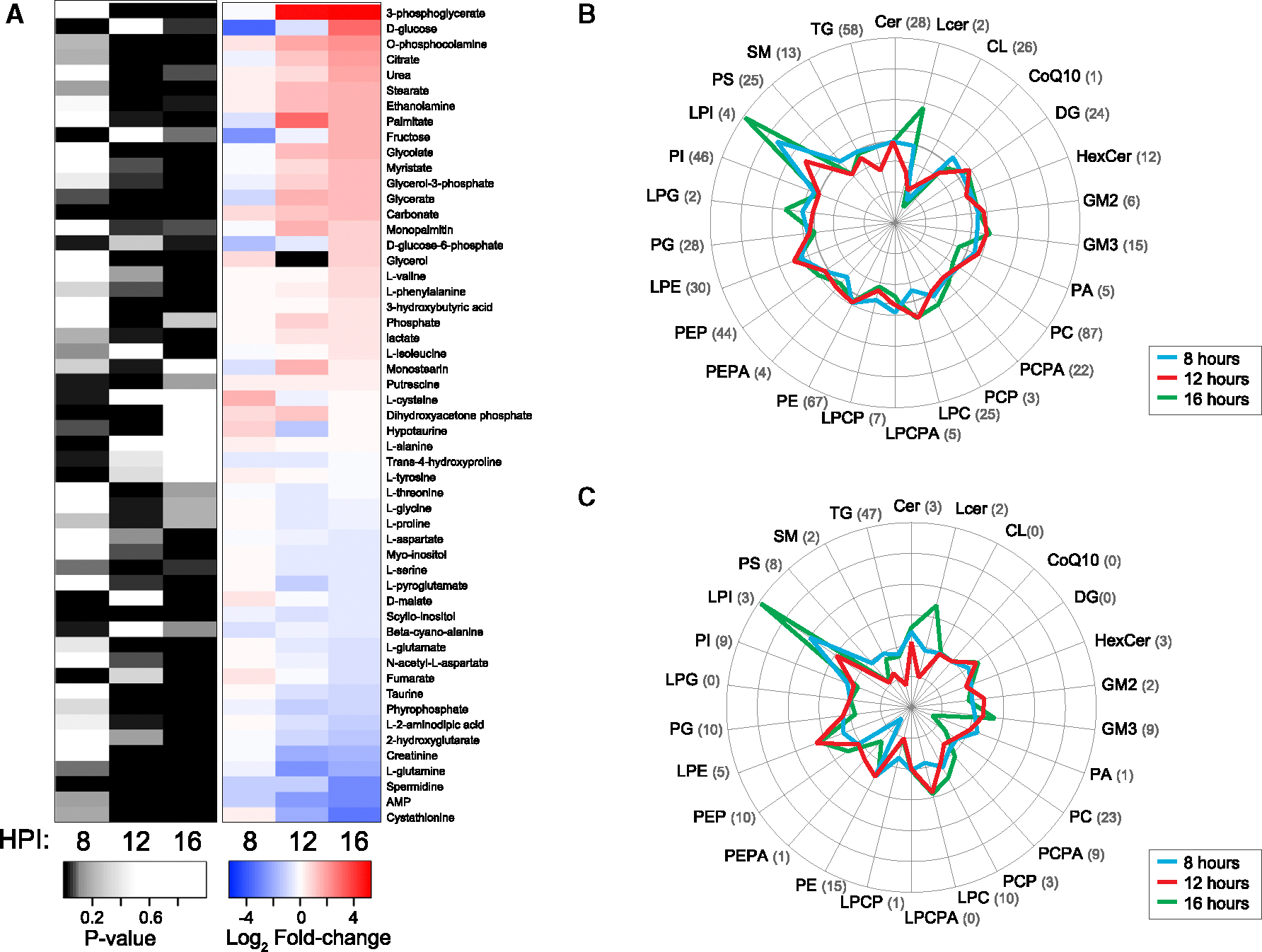
Summary diagram shows the metabolomic and lipidomic analyses of NiV infection (A) All metabolites exhibiting significantly altered levels (*p* ≤ 0.05) at 8, 12, or 16 hpi were included. (B) Average log_2_FCs of lipid sub-classes, up-regulation. (C) Average log_2_FCs of lipid sub-classes, down-regulation. (B and C) The numbers in parentheses indicate how many lipid species fall into each category.

Based on proteomics (Figures 2, 3, and 4), one of the clearest cellular changes during infection was the consistent increase in proteins associated with mitochondria, an organelle with crucial functions in processes such as immune responses, apoptotic signaling, and metabolism.^62,63^ While some viruses have been observed to target mitochondria for control over these processes during infections,^64,65^ much less is known about how metabolite and lipid profiles are altered, particularly in the case of acute infections, such as that of NiV. Samples from our paired mock and NiV infections were also processed for metabolomics and lipidomics analyses. As shown in Figure 5A, there were distinct metabolomic changes that occurred during infection, namely the fluctuation of several glycolytic products, fatty acids, and monoacylglycerols and the clear drop in several amino acids, such as L-glutamine (*p* = 0.012) and L-glutamate (*p* = 0.003), at 16 hpi. From lipidomics analyses, any significant changes in abundance were noted for all detected lipids (Figure 5B), grouped by sub-class. From this analysis, we identified a general trend of increased lysoceramides and lysophospholipids. Lysophosphatidylinositols, in particular, had the greatest increase of all lipids detected, with an average log_2_FC of 1.95 at 16 hpi.

Using Lipid Mini-On,^66^ an ontology and enrichment tool for lipidomics data, lipids that were statistically significantly regulated (*p* < 0.05) with a positive log_2_FC revealed that the sub-class phosphatidylethanolamine (PE) and glycerophospholipids with the fatty acid 16:1 were enriched at 8 hpi and sphingolipids, including ganglioside GM3, diacylglycerides (DGs), and lysophospholipids with C18 carbon chains, were enriched at 16 hpi (Table S3). Lipids that were statistically significant with a negative log_2_FC revealed that lipids containing saturated fatty acids were most depleted at both 8 and 16 hpi. Specifically, the fatty acid 24:0 (tetracosanoic acid) was enriched at 8 hpi, whereas 16:0 (palmitic acid) was enriched at 16 hpi (Table S3). At 8 hpi, glycerophosphocholines, including alkyl-acylglycerophosphocholines (PCOs), were enriched, and phospholipids with 16:0 and PCs with 14:0 were enriched at 16 hpi. At 12 hpi, triglyceride (TG) lipids with monounsaturated fatty acids (MUFAs), including 18:1, were enriched (Table S3). Most of the lipids containing 18:1 were associated with the TGs.

These findings highlight changes to specific sub-classes and species of lipids. In some cases, including human immunodeficiency virus, specific lipids such as phosphatidyl inositol are crucial during productive viral replication^67,68^ and were highlighted as some of the most significantly affected lipids during human Ebola virus disease.^69,70^ The biological significance of these shifts in metabolite and lipid profiles during NiV infection is unclear; however, the data reveal clear and strong trends offering an exciting new perspective into the life cycle of this virus and open many questions for future research.

### Overall model for metabolic changes in an infected cell using a multi-omics approach

**Figure 6.**
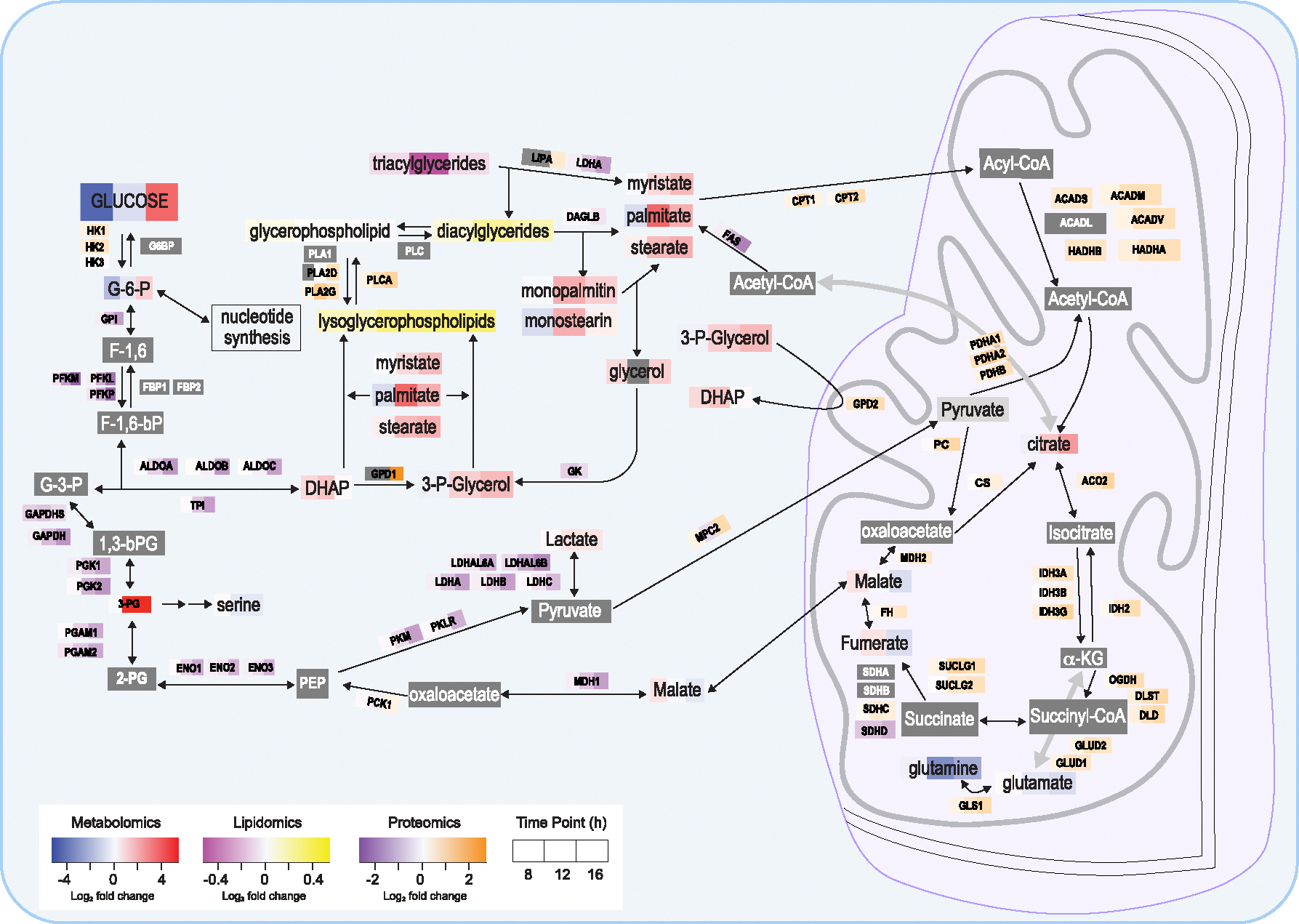
Overall model for metabolic changes in an infected cell using a multi-omics approach Data outputs from proteomics, lipidomics, and metabolomics were combined and used to construct a simplified model of cellular metabolic changes during NiV infection. Log_2_-transformed FCs are shown for each time point where possible. The absence of detection for an item is shown in dark gray; non-significant change at all time points is indicated in light gray.

To maximize the use of our findings, we summarized the major results from proteomics, metabolomics, and lipidomics analyses in a single diagram (Figure 6). From this analysis, a few fluctuation trends become clear. First, levels of glucose are significantly reduced after 8 h (*p* = 0.02; log_2_FC = −3.75), rebound toward normal levels at 12 hpi (*p* = 0.57; log_2_FC = −0.70), and increase substantially at 16 hpi (*p* = 0.04; log_2_FC = 3.13). The same fluctuation pattern, albeit weaker, was seen one step further in glycolysis for glucose-6-phosphate (G6P). This pattern was supported by our proteomic findings of concurrent, dramatic changes in all glycolytic enzymes except hexokinases such as hexokinase 1, which is up-regulated at both 12 (*p* < 0.001; log_2_FC = 0.77) and 16 (*p* < 0.001; log_2_FC = 0.69) hpi. Through lipidomics and metabolomics, it was also observed that TG levels were reduced during infection, with the lowest levels at 12 hpi, and that this reduction occurred along with increased levels of DGs, monoacylglycerides (monopalmitin and monostearin), fatty acids, glycerol, and glycerol-3-phosphate. Together, these markers may suggest an unexpected increase in TG catabolism during infection, although other interpretations are definitely possible, such as an increase in glycolysis.

### Overall model for post-transcriptional regulation of gene expression during NiV infection

One of the most striking phenotypes from all our analyses was the extreme inconsistency between proteomics and transcriptomics. First, the changes in abundance of many *individual transcripts* did not match the patterns of change for their associated proteins (Figure 4D). Additionally, this discrepancy was also clear when contrasting between the lists of *cellular processes* identified as changed, based on proteomics as compared to transcriptomics. These findings, along with proteomics analyses indicating reduced translation yet increased abundance for numerous clusters of functionally associated proteins (i.e., GO process and GO location), indicate the existence of differential post-transcriptional regulation of gene expression for specific protein groups. This is similar to findings from studies in cancer, which showed poor correspondence between the transcript and protein levels at the individual level as well as the pathway level.^71,72^ To aid in identifying contributing factors for these phenotypes, the proteomics data were summarized with a simplified model (Figure S1). As shown in this model, it is clear that ribosomal proteins and those involved in translation are generally reduced; however, further inhibition of cellular capability to support cytoplasmic translation may also be induced by reductions in the proteins involved in tRNA processing as well as rRNA and tRNA, but not mRNA, nuclear export. Additionally, proteins translated from nuclear RNA, translated in the cytosol, and imported into mitochondria, as well as those translated directly from mitochondrial RNA, exhibited generally increased abundances. The consistency and magnitude of increased mitochondrial protein abundance were unexpected and might indicate potential roles for this organelle in support of infection.

Another interesting finding with potential implications for the overall proteome and for the immune response to NiV infection was the consistent decrease in proteasomal subunit abundance. Interestingly, we also report consistent increases in major histocompatibility complex (MHC) class I proteins. Since proteasomal function is important during the infection of a cell for the processing of viral antigens, which are then presented to immune cells by MHC class I complexes,^73,74^ future studies are needed to understand the dynamic processing and turnover of these proteins and their effect on immune responses during NiV infection.

### F and G co-transfection displays an “infection-like” mitochondrial modulation in proteomics analysis

**Figure 7.**
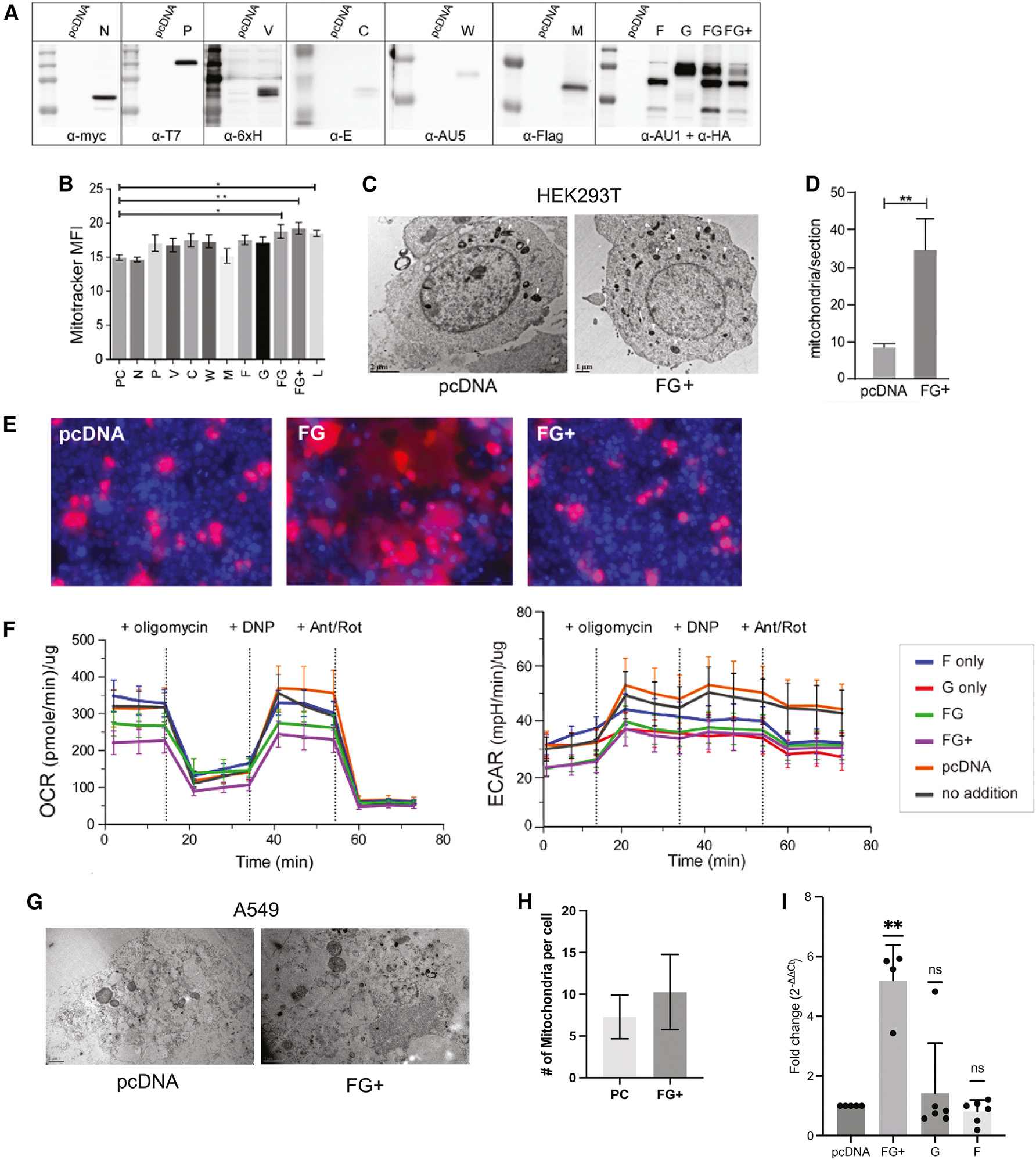
F and G co-transfection displays an infection-like mitochondrial phenotype in proteomics analysis (A) Expression of individual NiV proteins in HEK293T cells was detected by western blot analysis. (B) Mitochondrial content of infected and control HEK293T cells was measured using MitoTracker staining. (C) Transmission electron micrograph of F- and G-transfected HEK293T cells. Scale bars, 2 μm (left image) and 1 μm (right image). White triangles indicate mitochondria. (D) The number of mitochondria and mitochondria-like structures was counted and analyzed using ImageJ software. t-test *p* < 0.01. (E) Fluorescence imaging of fusion formation at 24 hpi. HEK293T cells were transfected with F, G, FG, pcDNA, and RFP-HA. HR2 peptide is included in the FG+ wells to block syncytia formation. Red is RFP-HA (cytosol) and blue is Hoechst (nuclei) staining. (F) Respirometry analysis on F- and G-transfected cells. Seahorse assay with reagents specific for oxygen consumption rate (left) was performed, with a secondary readout of extracellular acidification rate (right), indicating no increase in the metabolic rate. (G) Mitochondrial content of FG transfected into A549 cells. (H) The number of mitochondria in cells was imaged via TEM, counted, and then analyzed using GraphPad Prism. A t test yielded a *p* value of 0.0757. (I) qPCR analyses showing FC (2^−ΔΔCt^) compared to the empty vector control (pcDNA). Statistical analyses for qPCR were performed using one-way ANOVA (****p < 0.0001).

To determine the impact of NiV glycoproteins on the host, HEK293T cells were infected with the NiV Malaysia strain at MOI 1 under BSL-4 conditions. We performed a proteomics analysis on the combined time courses by applying two criteria to refine the data: a peptide score ≥ 4 and enrichment over uninfected samples (Table S4). We further divided the refined data into subcellular organelles using Cytoscape^75^ key term functions. Among the analyzed pathways, mitochondria displayed the highest number of proteins, and we chose mitochondria as a clear pathway affected to validate our study (Table S5). To validate these omics findings, we measured MitoTracker, a dye that stains live mitochondria, in the presence of NIV genes individually or in combination after transfection via flow cytometry (Figures 7A and 7B). Interestingly, the transfection of two viral envelope proteins, F and G, was sufficient to observe an increase in MitoTracker fluorescence. Further, we obtained electron micrographs of FG-co-transfected and control HEK293T and A549 cells. We observed an increase in the number of mitochondria in cells transfected with FG plasmids compared to vector control (Figures 7C and 7D). To compare this phenotype to the infection data, we performed another proteomics analysis on HEK293T cells transfected with envelope genes either individually or combined (Table S8). We applied the same criteria and analysis as above. This method has generated another list of mitochondrial proteins for transfection data. We compared the two datasets and observed that they display an extensive similarity (Table S5). F and G co-transfection yielded higher numbers (84) of mitochondrial proteins when compared to F-only (11) and G-only (11) transfections. Remarkably, 88% of the proteins in this list appeared in the infection-proteomics list. To address the potential effect of fusion-related events, if any, we included the fusion inhibitor HR2 for the whole duration of MitoTracker staining experiments to control for syncytial cells. We still observed that MitoTracker staining was significantly increased when compared to the vector control and concluded that this increase was independent of syncytia formation (Figures 7B and 7E).

To address if the increase in mitochondrial content translated to an increase in mitochondrial function, we performed cellular respirometry assays on transfected HEK293T cells. To investigate the function of the increased mitochondria, we used specific reagents (oligomycin, 2,4-dinitrophenol [DNP], and antimycin A/rotenone [Ant/Rot]) to measure the mitochondrial respiratory capacity. Interestingly, the cellular oxygen consumption rate (OCR)—a proxy of mitochondrial electron transport chain (ETC) function—did not mirror the increase in mitochondrial content observed from MitoTracker staining (Figure 7F). The discrepancy between the increased mitochondrial content and decreased OCR may suggest that NiV F and G are capable of increasing the number of mitochondria in a non-canonical manner. Moreover, it may be speculated that the presence of NiV F and G in cells induces cellular stress; thus, the cell attempted to overcome stress by increasing the number of mitochondria, yet the function was not correlated.

To corroborate our results obtained in HEK293T cells in a more relevant cell line, we subsequently performed our mitochondria validation experiments in A549 cells (*Homo sapiens*, lung epithelial cells), wherein we observed a similar pattern in increased mitochondrial proteins. We noted a differential transfection efficiency across the two cell lines, and thus we added different methodologies to assay the mitochondrial content, including electron microscopy (EM) (Figures 7G and 7H) and qPCR (Figure 7I). These data corroborate our initial findings regarding a trend or statistical significance in enhanced mitochondria in the different and biologically relevant lung epithelial cell type A549 (Figures 7G–7I).

## DISCUSSION

Over the last few decades, major advances in mass spectrometry software, instrumentation, and methods have dramatically improved approaches to understanding protein interactions, post-translational modifications, and changes in abundance.^76,77^ Here, we report dramatic changes to the transcriptional, proteomic, and lipidomic profiles of cells infected with the deadly NiV (Figure 1). This study offers comprehensive and descriptive analyses of cellular changes upon infection of NiV using omics, which will serve as a crucial dataset for further research. Focusing particularly on proteomic changes, we find that infection may potentially alter protein levels at multiple points after transcription, including mRNA splicing and translation, as well as RNA and protein degradation. While these findings suggest a global decrease in protein expression, this reduction appears to be highly dependent on subcellular protein localization and primarily affects cytoplasmic proteins. The most outstanding example of a difference from the global decrease was the large and consistent increase in mitochondrial proteins (Figures 2, 3, and S1). In addition to syncytia formation, infected cells may exhibit large cytoplasmic lipid droplets, mitochondrial swelling, and vacuolization.^28,78^ While each of these characteristics has ties to stress responses and programmed cell death, their importance, origins, and timing in the context of NiV infection are unknown. In fact, relatively little is known about the cellular changes that occur during a henipavirus infection, including the cellular innate immunity, stress, and programmed death responses induced. Understanding such features is key to understanding pathogenesis during NiV infections.

While several viruses have been shown to interact with individual mitochondrial proteins or complexes to influence their involvement in metabolism, cell death, and immunity, our report of highly consistent increases in mitochondrial proteins is, to our knowledge, novel for infections with negative single-stranded viruses (order Mononegavirales). Importantly, very little has been uncovered concerning functional interactions between henipaviruses and mitochondria.^64,65^ Our results have shown an increase in mitochondrial content that is independent of syncytia formation and the respirational state of the cell. This phenotype can be recapitulated only when two envelope proteins are co-expressed. Envelope proteins are transmembrane proteins, and plasma membrane trafficking after their synthesis in the ER is the most common pathway taken by these proteins during infection. The membrane interaction sites between mitochondria and the ER are studied in detail and have been shown to be niches for modulation between membrane-contacting organelles.^77,79–82^ However, the incorporation of viral glycoproteins in these contact sites is not well characterized. Moreover, functional or structural integration of viral proteins into these highly specific niches needs to be addressed, as we observed an increase in mitochondria yet no functional increase in mitochondrial respiration. We speculate that the increase in mitochondrial levels is in response to the viral infection stress but that functionally, the mitochondrial system may be defective, such that it cannot boost or compensate the respiratory capacity. Indeed, this has been shown previously in cases of mitochondrial dysfunction where the cell boosts mitochondrial levels in an attempt to overcome mitochondrial dysfunction, yet the mitochondria are still defective.^83,84^ As we observed increased glycolysis-related proteins from our proteomics, we speculate that NiV utilizes alternative metabolic pathways, such as glycolysis, as an alternative source of energy.

A recent study of the closely related HeV suggested that cell death may be inhibited during henipaviral infection of HEK293T cells.^45^ Increased reactive oxygen species (ROS) production and other proteomic changes likely contribute to changes to mitochondrial morphology and biogenesis later during infection. The increase in mitochondrial content independent of an increase in respirational function strongly suggests NiV-mediated alternative pathways that may include calcium signaling and mitochondrial dynamics. Indeed, healthy mitochondrial populations are thought to be maintained by continuous cycles of mitochondrial fusion and fission.^85^ During NiV infection, the proteins involved in mitochondrial fusion, OPA1 and mitofusion-1, were increased significantly at 16 hpi (log_2_FC = 1.11 and 1.04, respectively), with OPA1 also showing a trend toward enrichment at 8 and 12 hpi. On the contrary, a protein that plays a role in mitochondrial fission, Dynamin-1-like protein, exhibited statistically significant decreases in abundance from 8 to 16 hpi (8/12/16 hpi, log_2_FC = −0.34/−1.12/−1.94). Whether these changes are virally or cellularly driven is unclear; any ensuing mitochondrial change might have important roles in pathology and may explain a previous report of enlarged mitochondria during NiV infection of porcine neuronal cells.^78^

A previous study identified interactions between several products of the P gene (P, V, and W) and the prp19 spliceosomal complex.^43^ Interestingly, we found that several of these identified factors, including prp19 itself, are significantly up-regulated during infection. As was previously suggested, these interactions may be important for virus-driven apoptotic avoidance or for a more general influence over RNA splicing. In support of infection-associated changes to RNA splicing, our REVIGO analysis identified RNA processing (*p* = 10^−82^) and mRNA processing (*p* = 10^−59^) as the two most significantly enriched GO processes based on up-regulated proteins (Figure 3B). Similarly, while we identified an increased expression of p53 at 12 (*p* < 0.001; log_2_FC = 1.11) and 16 (*p* < 0.01; log_2_FC = 1.30) hpi, our Reactome pathway analysis suggested a trend of reduced expression for proteins associated with apoptosis^86^ (Table S3). This finding is also supported by our lipidomics data and specifically by the decrease in ceramide abundance during NiV infection. The potential absence of apoptosis is also consistent with a recent study of HeV, where infection in reservoir bat cells, but not human cells, led to the induction of apoptosis.^45^ Based on infections with other types of viruses, the inhibition of apoptosis during NiV infection may serve to blunt the immune response and further support viral spread through cell-cell fusion.^87–89^

Another finding from our Reactome analysis was that proteins associated with small interfering RNA (siRNA) biogenesis were substantially down-regulated at 12 and 16 hpi (Table S2). Based on the antiviral roles of siRNAs, this could be another possible point of viral influence on cellular protein expression during infection and/or may serve as a point of inhibition of the antiviral response.^90–93^ Supporting these possibilities, the NiV M was recently identified as a binding partner of the Dicer1 protein, which plays a central role in the formation of microRNAs and siRNAs.^43,94^ A functional understanding of NiV-siRNA interactions and their importance during infection remains to be elucidated.

In the hope of validating and deepening the knowledge gained from proteomics, we also conducted lipidomics and metabolomics analyses of NiV infection. In agreement with the literature, we observed cyclical fluctuation of lipids during our time points, possibly attributed to the viral hijacking of host lipid metabolism.^95–97^ In the current phase, our focus from metabolomics data is observations rather than drawing conclusive interpretation. This dataset serves as the foundational basis for upcoming investigations. We report highly consistent decreases in abundance for proteins involved in glycolysis, whereas protein expression of citric acid cycle enzymes was elevated. Since many of the other glycolytic products past G6P were not detected, this may suggest that glucose and G6P were primarily targeted for nucleotide synthesis to support viral replication. A major exception to this seemingly broad fluctuation of glycolysis was that hexokinase, a key metabolic regulatory enzyme and the first enzyme of glycolysis, is up-regulated in expression at both 12 and 16 hpi (Figure 6). We speculate that the clear increases in the abundance of hexokinases may serve the purpose of adding charge to glucose, thus keeping it from leaving the cell so that it may be used during infection.^98^ Further, phosphorylated glucose may also be shunted from glycolysis and instead utilized for nucleotide synthesis to sustain viral replication. Interestingly, 3-phosphoglycerate (3-PG) was observed to have increased abundance, yet its downstream glycine and serine were not. Given the current literature’s observation of serine metabolism regulating immune cell function, and glycine’s ability to halt virus invasiveness, we may infer that although the 3-PG is increased, its downstream amino acids are restricted to foster efficient viral infection.^99,100^ At this point, we cannot conclude whether this phenomenon is due to NiV redirecting amino acids to be used for nucleotide synthesis or other cellular modulation relating to its infection.

The reduction in seleno-protein expression and seleno-amino acid metabolism supports the likely involvement of ROSs and their release in inflammation during NiV infection.^101^ Similar findings of faulty ROS removal in cells infected with human respiratory syncytial virus, a pathogen distantly related to NiV, as well as other respiratory viruses have been tied to the high levels of inflammation that occur during infection and contribute to pathology.^102,103^ Among cellular antioxidant proteins identified as reduced in abundance were those involved in the synthesis and continued activity of glutathione, a crucial cellular antioxidant, including glutathione synthetase (12/16 hpi, log_2_FC = −0.87/−1.03) and glutathione reductase (12/16 hpi, log_2_FC = −0.94/−1.41). Numerous other enzymes important for directly maintaining safe ROS concentrations were also reduced: superoxide dismutase (12/16 hpi, log_2_FC = −0.96/−1.74), peroxiredoxin-1 (12/16 hpi, log_2_FC = −0.90/−1.44), and thioredoxin (12/16 hpi, log_2_FC = −1.29/−2.24). We thus suggest that ROSs accumulate during NiV infection due to drastic reductions in antioxidant proteins and molecules, leading to greatly exacerbated tissue damage.

NiV is known to inhibit activation of the type I interferon response through several means.^33,104^ Similar to the influenza NS1 protein, the V protein of NiV has been shown to inhibit the RIG-I pathway through direct interaction and inhibition of TRIM25, which is crucial for RIG-I activation.^32,105^ We identified statistically significant reductions in the abundance of TRIM25 (8/12/16 hpi, log_2_FC = −0.24/−0.99/−2.12), as well as another key downstream component of the RIG-I pathway, TBK1 (8/12/16 hpi, log_2_FC = −0.47/−1.35/−2.18). REVIGO analyses for 16 hpi identified proteins involved in antigen processing and presentation via MHC class II, and the interleukin-12-mediated signaling pathway is down-regulated. Interestingly, proteins involved in MHC class I peptide presentation were generally increased in abundance, particularly at 12 hpi; however, proteasomal subunits were drastically and consistently reduced (Figure S1). It remains to be seen how these changes in abundance might affect MHC class I function and immune response. Whether any of these processes are further targeted by specific function(s) of NiV proteins remains to be determined and of future interest.

In summary, NiV infection of mammalian cells was analyzed with a multi-omics approach across four time points to best understand major cellular changes. Multiple processes, including gene expression, metabolism, and immune responses, were all affected over the course of infection. Among the most unexpected findings was the highly significant up-regulation of mitochondrial proteins and potential changes to mitochondrial dynamics and organization, offering new insights into NiV pathogenicity and the potential importance of mitochondria in the NiV life cycle. Notably, changes in mitochondria were validated by multiple assays. Additionally, we report a stark inconsistency between transcriptomic and proteomic changes during infection, which indicates the potential post-/co-transcriptional influence of NiV on the proteome, further supported by a recent report of interactions between NiV proteins and both RNA splicing and RNAi machineries.^43^ This same disparity has been documented in other studies, providing additional examples of the limited correlation between transcripts and proteins upon viral infection.^106–109^ The field has acknowledged these disparities, utilizing them to identify post-transcriptional degradation targets of viruses or novel viral mechanisms for further investigation into virus-induced degradation across various levels of host cellular products.^110,111^ In summary, we have described the first look with such depth into the many intracellular changes that occur during a henipavirus infection. The findings described here serve to identify novel mechanisms of henipaviral and cellular actions, as well as to provide a contextual foundation for their future studies.

### Limitations of the study

Due to restrictions associated with the accessibility of BSL-4 pathogens and facilities, besides our NiV infection studies, we employed an additional co-transfection model using NiV protein expression plasmids. These experiments attempted to further investigate the potential roles of specific viral proteins during NiV infection. While the proteomic datasets from naive NiV infection vs. the transfection model exhibited substantial similarities, it is likely that there are underlying mechanistic differences between the infection and transfection studies, particularly since it is impossible to express all viral proteins at identical expression ratios as in real viral infections and in every cell. We observed inconsistencies between transcriptomics and proteomics data during natural NiV infection, a phenomenon commonly observed in omics studies. These discrepancies highlight the complexity of the host response to viral infection and the need to further investigate such differences. The contribution of specific genes to infection-like phenotypes needs additional study. For example, any potential role of F and G in enhancing mitochondrial proteins needs further confirmation. Weadditionally acknowledge that the counting of mitochondria from transmission electron microscopy (TEM) images can be subjective. Despite these limitations, our study provides valuable insights into the intracellular modulations of host cells upon NiV infection. Importantly, our work highlights the integration of transcriptomics, proteomics, lipidomics, and metabolomics data to investigate the cellular processes that result from henipaviral infections.

## RESOURCE AVAILABILITY

### Lead contact

Further information requests for resources and reagents should be directed to and will be fulfilled by the lead contact, Hector C. Aguilar (ha363@cornell.edu).

### Materials availability

This study did not generate new unique reagents.

### Data and code availability

The raw data have been deposited at Gene Expression Omnibus (https://www.ncbi.nlm.nih.gov/geo/) and MassIVE (https://massive.ucsd.edu) and are publicly available as of the date of publication. All data reported in this paper will be shared by the lead contact upon request.

## STAR★METHODS

### EXPERIMENTAL MODEL AND STUDY PARTICIPANT DETAILS

For this study, we used two immortalized human cell lines: HEK293T and A549. Both cell lines were obtained from and authenticated at American Type Culture Collection (ATCC). Both cell lines tested negative for mycoplasma contamination within our lab. Both cell lines were grown at 37°C in 5% CO_2_ in Dulbecco’s Modified Eagle Medium (DMEM) supplemented with 10% fetal bovine serum (FBS). No human subjects/samples were used in the study.

#### Biosafety statement

All work with infectious Nipah virus was conducted in the biosafety level 4 (BSL-4) laboratory located in the Integrated Research Facility of the Rocky Mountain Laboratories, National Institute of Allergy and Infectious Diseases (NIAID), National Institutes of Health (NIH). Sample inactivation and removal was performed according to established standard operating protocols approved by the Institutional Biosafety Committee (IBC) and certified by the Division of Select Agents and Toxins (DSAT).

#### Cells and reagents

HEK293T cells were obtained from ATCC and used for all experiments, both at biosafety levels 2 and 4. These cells were grown in Dulbecco’s Modified Eagle Medium supplemented with 10% Fetal Bovine Serum and 1% Penicillin/Streptomycin (Gibco) and grown at 37°C with 5% CO_2_. A549 cells used in some experiments were grown in Dulbecco’s Modified Eagle Medium supplemented with 10% Bovine Calf Serum, 10mM HEPES pH 7.5 and 1% Penicillin/Streptomycin (Gibco) and grown at 37°C with 5% CO_2_.

#### Viral strain

The Malaysia strain of NiV was used for BSL-4 experiments described. At the time of planning the logistics of this study (several years ago), we didn’t have access to the Bangladesh strain. Further, Nipah virus is a select agent and BSL-4 agents are highly regulated and require longer preparation time between the institutions. These factors led to our use of the Malaysia strain, which still shares high sequence homology (>95%) with the Bangladesh strain. Therefore, we believe our results to be relevant to either viral strain and likely beyond Nipah virus to other henipaviruses.

### METHOD DETAILS

#### NiV infection, irradiation, and sample preparation for omics analyses

For each of four time-points, ten 60mm plates and ten 6-well plates were seeded with HEK293T cells. After seeding, each group of ten plates was divided into two groups of five. One group was left uninfected (mock) and the other was infected with NiV, Malaysian strain, at a multiplicity of infection (MOI) of 1. After 4, 8, 12, and 16 hpi, cells from each 60 mm dish were collected with PBS and irradiated at 10 Mrads to inactivate NiV. The irradiated cells were then prepared for proteomic, metabolomic, and lipidomic analyses. Similarly, cells in the 6-well plates were treated with TRIzol for extraction of RNA to be analyzed by microarray. Prior to harvest for each experiment and time-point, representative images of infected or mock-infected cells were taken with a BioRad Zoe light microscope and prepared for publication using Fiji software.

#### Transcriptomics

Samples were sent to Arraystar (Rockville, MD) for RNA extraction and microarray analysis with the Agilent Human 4 × 44K gene expression array. Total RNA from each sample was quantified using the NanoDrop ND-1000 and RNA integrity was assessed by standard denaturing agarose gel electrophoresis. The sample preparation and microarray hybridization were performed based on the manufacturer’s standard protocols. Briefly, total RNA from each sample was amplified and transcribed into fluorescent cRNA using the manufacturer’s Agilent’s Quick Amp Labeling protocol (version 5.7, Agilent Technologies). The labeled cRNAs were hybridized onto the Whole Human Genome Oligo Microarray (4 × 44K, Agilent Technologies). After washing the slides, the arrays were scanned by the Agilent Scanner G2505C. Background correction was carried out on microarray samples using the maximum likelihood estimation for the normal-exponential convolution model,^112^ with an offset of 50, as implemented in Bioconductor’s^113^ *limma* package.^114^ Samples were then normalized using quantile normalization and outliers were detected using intensity distribution and a boxplot graph followed by hierarchical clustering and PCA analysis of expression profiles using the *MVA* package.^115^

#### Multi-omics sample extraction

All chemicals and solvents are from Sigma-Aldrich (St. Louis, MO) unless otherwise stated. The infected cell pellets were processed to extract proteins, metabolites, and lipids utilizing the MPLEx method.^116^ Briefly, each pellet was brought up to a volume of 200 μL with Milli-Q water, and 1 mL of an ice-cold 2:1 Chloroform:Methanol (C:M) solution was added to each sample and vortexed for 30 s followed by storage on ice. The three visible layers of proteins, metabolites, and lipids were each transferred to separate vials and the protein pellet was washed with 1 mL of ice-cold methanol and dried. The lipidomic (lower, chloroform) and metabolomic (upper, methanol) layers were dried in a SpeedVac (ThermoFisher Scientific, Pittsburgh, PA). The lipidomic layer was reconstituted in 500 μL 2:1 C:M while the metabolomic layer was kept dry and both were stored at −80°C.

The protein pellet was digested in a manner similar to the MPLEx method with two exceptions. After the pellets were resuspended with 100 μL of 1 mM MgCl_2_ in 10 mM Tris-HCl, 2 μL of 2.5U/uL benzonase was added to each sample and then stored overnight at 4°C. An additional 2 μL of 2.5U/uL benzonase was added to each sample and incubated at 37°C for 30 min, 850 rpm in a thermomixer (Eppendorf, Hauppauge, New York). Urea was added to each sample to an 8M concentration and a bicinchoninic acid (BCA) protein assay was performed. The samples were then reduced and alkylated with 5 mM dithiothreitol and 40 mM iodoacetamide. Samples were then diluted 8-fold with 50 mM ammonium bicarbonate, and CaCl_2_ was added to achieve a concentration of 1mM per sample. The samples were digested with trypsin (Affymetrix, Santa Clara, CA) for 3 h and desalted using a C18 solid phase column (Phenomenex, Torrance, CA) with elution in 400 mL of 80% acetonitrile (ACN) and 0.1% trifluoroacetic acid (TFA). Samples were then dried and reconstituted in 50 μL of 5% ACN. Protein concentrations were determined by a second BCA assay and samples were normalized to a final peptide concentration of 0.1 μg/μL.

#### Proteomics

Peptide samples (5 μL) were analyzed by LC-MS/MS using a Waters nano-Acquity M-Class dual pumping UPLC system (Milford, MA) configured for on-line trapping at 5 μL/min for 8 min followed by gradient elution through a reversed-phase analytical column at 300 nL/min. Columns were packed in-house using 360 μm o.d. fused silica (Polymicro Technologies Inc.) and contained Jupiter C18 media (Phenomenex) in 5 μm particle size for the trapping column (100 μm i.d. × 4 cm long) and 3 μm particle size for the analytical column (75 μm i.d. × 70 cm long). Mobile phases consisted of (MP-A) 0.1% formic acid in water; and (MP-B) 0.1% formic acid in acetonitrile with the following gradient profile (min, % MP-B): 0, 1; 2, 8; 20, 12; 75, 30; 97, 45; 100, 95; 110, 95.

MS analysis was performed using a Q-Exactive HF mass spectrometer (Thermo Scientific, San Jose, CA). Electrospray emitters were prepared in-house using 150 μm o.d. × 20 μm i.d. chemically etched fused silica (Kelly et al., 2006) and subsequently attached to the column using a metal union and coupled to the mass spec via a custom built nanospray source. Electrospray voltage (2.2 kV) was applied at the metal union providing ions to the heated (325C) ion transfer tube entrance of the MS. Data acquisition was started 20 min after the gradient began and continued for a total acquisition time of 100 min. Precursor MS spectrum were acquired from 400 to 2000 *m/z* at resolution 60k (AGC target 3e6, max IT 20 ms) followed by data-dependent MS/MS spectra of the top 12 most abundant ions from the precursor spectrum with an isolation window of 2.0 *m/z* and at a resolution of 15k (AGC target 1e5, max IT 200 ms) using a normalized collision energy of 30 and a 45 s exclusion time.

LC–MS/MS raw data were converted into dta files using Bioworks Cluster 3.2 (Thermo Fisher Scientific), and the MSGF+ algorithm was used to search MS/MS spectra against the Human Uniprot 2016–04-13 database with 20154 entries and against Uniprot entries for individual Nipah virus proteins. The key search parameters used were ±20 ppm tolerance for precursor ion masses, +2.5 Da and −1.5 Da window on fragment ion mass tolerances, MSGF+ high resolution HCD scoring model, no limit on missed cleavages but a maximum peptide length of 50 residues, partial or fully tryptic search, variable oxidation of methionine (15.9949 Da), and fixed alkylation of cysteine (carbamidomethyl, 57.0215 Da). The decoy database searching methodology was used to control the false discovery rate (FDR) at the unique peptide level to <1% and subsequent protein level to <0.5% (% FDR = ((reverse identifications*2)/total identifications)*100). Identification and quantification of the detected peptide peaks were performed by using the label-free Accurate Mass and Time (AMT) tag approach.^117,118^ Briefly, an AMT tag database was created from the MS/MS results, and in-house developed informatics tools (including algorithms for peak-picking and determining isotopic distributions and charge states, which are publicly available at https://omics.pnl.gov/software) were used to process the LC-MS data and correlate the resulting LC-MS features to an AMT tag database. Further downstream data analysis incorporated all possible detected peptides into a visualization program, VIPER,^119^ to automatically correlate LC-MS features to the peptide identifications in the AMT tag database. The resulting post-VIPER matching proteomics data were filtered to achieve an absolute average mass error of 1.08 ppm and an absolute average net elution time error of 0.16%.

#### Metabolomics

The extracted metabolite samples, flash frozen then thawed in extraction solution, from the infected cells using MPLEx protocol^49^ were shipped to PNNL for metabolomics analysis. The extracts were chemically derivatized for GC-MS analysis as reported previously.^120^ Briefly, 20 μL of methoxyamine in pyridine (30 mg/mL) were added to each sample, followed by vortexing for 30 s and incubation at 37°C with generous shaking (1,200 rpm) for 90 min to derivatize hydroxyl and amine groups to trimethylsilyated forms, 80 μL of N-methyl-N-(trimethylsilyl) trifluoroacetamide with 1% trimethylchlorosilane were then added to each vial, followed by vortexing for 10 s and incubation at 37°C with shaking (1,200 rpm) for 30 min. Samples were analyzed by Agilent GC 7890A coupled with a single quadrupole MSD 5975C (Agilent Technologies), and the samples were blocked and analyzed in random order. An HP-5MS column (30 m × 0.25 mm × 0.25 μm; Agilent Technologies) was used for global metabolomics analyses. The sample injection mode was splitless, and 1 μL of each sample was injected. The injection port temperature was held at 250°C throughout the analysis. The GC oven was held at 60°C for 1 min after injection, and the temperature was then increased to 325°C by 10 °C/min, followed by a 10-min hold at 325°C. MS data were collected over the mass range 50–550 *m/z*. A mixture of FAMEs (C8-C28) was analyzed once per day together with the samples for retention index alignment purposes during subsequent data analysis. GC-MS raw data files were processed using the Metabolite Detector software, version 2.5.2 beta.^121^ Retention indices of detected metabolites were calculated based on the analysis of the FAMEs mixture, followed by their chromatographic alignment across all analyses after deconvolution. Metabolites were initially identified by matching experimental spectra to a PNNL-augmented version of Agilent Fiehn database, containing spectra and validated retention indices for over 850 metabolites. Metabolite identifications were manually validated to reduce deconvolution errors during automated data-processing and to eliminate false identifications. The NIST 14 GC-MS library was also used to cross validate the spectral matching scores obtained using the Agilent library and to provide identifications of unmatched metabolites.

#### Lipidomics

The extracted lipid samples were shipped to PNNL for lipidomics analysis. Extracted lipids were analyzed by LC-MS/MS using a Waters NanoAcquity UPLC system interfaced with a Velos Orbitrap mass spectrometer (Thermo Scientific, San Jose, CA) as outlined in Dautel et al., 2017.^122^ Briefly, lipid extracts were reconstituted in 200 μL of methanol, and 7 μL of each sample was injected and separated over a 90-min gradient elution (mobile phase A: ACN/H_2_O (40:60) containing 10 mM ammonium acetate; mobile phase B: ACN/IPA (10:90) containing 10 mM ammonium acetate) at a flow rate of 30 μL/min. Samples were analyzed in both positive and negative ionization (full scan range of 200–2,000 *m/z*) using HCD (higher-energy collision dissociation) and CID (collision-induced dissociation) on the top 6 most abundant ions to obtain high coverage of the lipidome.

Confident lipid identifications were made by using LIQUID, which enables the examination of the tandem mass spectra for diagnostic ion fragments along with associated hydrocarbon chain fragment information. In addition, the isotopic profile, extracted ion chromatogram, and mass measurement error of precursor ions were examined for each lipid species. To facilitate quantification of lipids, a reference database for lipids identified from the MS/MS data was created, containing the lipid name, observed *m/z*, and retention time. Lipid features from each analysis were then aligned to the reference database based on their *m/z* and retention time using MZmine 2. Aligned features were manually verified and peak apex intensity values were exported for subsequent statistical analysis. Positive and negative ionization data were analyzed separately at all stages.

#### Expression plasmids and transfection of HEK293T and A549 cells

The codon optimized Nipah virus genes, epitope tags, length, and molecular weights are listed in Table S6. L protein, which has no tag because of large size was included in only flow cytometry experiments and excluded in the rest of the experiments. Epitope tagged over-expression plasmids (1μg total) were transfected for 4 h with polyethylene-imine (PEI, Polysciences) or Lipofectamine 2000 (ThermoFisher Scientific) using empty pcDNA3.1as filler DNA into 3.10^5^ HEK293T cells growing in 24-well plates or A549 cells growing in 6-well plates, followed by addition of complete media until collection at 24 h. For F and G co-transfections 3:1 DNA ratio was empirically determined to observe fusion events. For large scale transfections, five 15-cm^2^ plates were used in each condition. 30 μg plasmid DNA per plate was transfected using 4:1 ratio instead.

#### Quantification of mitochondrial content by flow cytometry

3.10^5^ log phase HEK293T or A549 cells were plated on poly-lysine treated 24-well plates and transfected for 4 h with individual Nipah virus genes and RFP-HA plasmid. In order to inhibit fusion events, in-house prepared HR2 peptide (KVDISSQISSMNQSLQQSKDYIKEAQRLLDTVNPSL) was included in transfection solution all the time at 5 nM concentration. HR2 peptide was serially diluted and tested in NiV F and NiV G transfected cells. We chose 5 nM, a concentration that gives >90% inhibition, and peptide was present throughout the whole experiment.^123–125^ Briefly, the Fmoc protected peptide on rink-amide resin was validated using high resolution mass spectrometry and HPLC. Fmoc removal (deprotection) was performed using 20% piperidine in dimethylformamide (DMF) at room temperature. The process was monitored using Kaiser Test (Reagent A: 0.4 mL, 1mM KCN in 20 mL pyridine, Reagent B: 0.5 g ninhydrin/10mL Ethanol). Deprotected peptide was cleaved using 90/2.5/2.5/2.5/2.5 TFA/EDTA/TIS/Thioanisole/H20 under Ar blanket for 2 h at room temperature. Peptide was then purified using reverse phase HPLC using a C18 column (Waters X-Bridge BEH130, 10 × 250 mm, 5μm). Eluates were concentrated, peptide-chloride salts lyophilized to remove residual TFA. Peptide identity was verified using MALDI-TOF mass spectrometry. Biological function was verified empirically using cell-cell fusion inhibitions on F (fusion gene) and G (attachment gene) co-transfected HEK293T cells.

At 23 h post-transfection, cells were stained with 100 nM MitoTracker FM (M7514, Invitrogen) at 37°C according to manufacturer’s instructions. Cells were collected by pipetting up and down, and washed thrice with PBS buffer [1% bovine serum albumin (BSA) in PBS] at 250×g, 10 min, at 4°C. Final cell suspensions were run in Guava Easycyte 8HT flow cytometry. Transfected cells were gated on red channel (RFP) and mitochondrial content was measured using green channel (MitoTracker green). Green signal coming transfected cells are normalized to green signal from non-transfected cells.

#### Quantification of mitochondrial DNA by real-Time PCR

A549 cells were transfected as described above, with empty vector (pcDNA), NiV F and G (with HR2 peptide), NiV G alone, or NiV F alone using Lipofectamine 3000. After 30 h post-transfection, total cellular DNA was extracted using SpeeDNA Isolation Kit according to the manufacturer’s instructions (ScienCell, #MB6918). RT-PCR was used to determine changes in ratio of mitochondrial DNA (mtDNA) to nuclear DNA (SCR, single copy reference) using ScienCell’s qPCR Assay Kit (ScienCell, #8948). All reactions were performed in 96-well plates using the Applied BiosystemsTM 7500 Real-Time PCR Systems. Reference genomic DNA with known copy of mitochondrial DNA was used as an internal control for primers. All qPCR reactions were performed in duplicates, and Ct values were averaged. Amplification was performed under following conditions: denaturation for 10 min at 95C, followed by 32 cycles of denaturation for 20 s at 95C, annealing for 20 s at 52C, and extension for 45 s at 72C. The fold change (2^−ΔΔ^Ct) was calculated using ΔCt (mtDNA) = Ct (mtDNA of NiV FG) – Ct (mtDNA of pcDNA), ΔCt (SCR) = Ct (SCR of NiV FG) – Ct (SCR of pcDNA), then ΔΔCt = ΔCt (mtDNA) - ΔCt (SCR) to assess relative mtDNA copy number of target sample NiV FG to reference pcDNA sample = 2^−ΔΔ^Ct. The relative fold change values are reported as mean ± standard deviation (SD).

#### Western Blot analysis

Nipah virus genes and pcDNA3.1 plasmid (filler) were used to transfect the log phase HEK293T cells. 3.10^5^ cells were plated on polylysine coated 24-well plates and transfected with 1 μg total DNA using PEI. At 24 h post-transfection cells were collected by pipetting up/down in the wells and transferred into cold 1.5 mL tubes. Cell suspensions were spun at 500g for 10 min at 4C. Pellets were washed with PBS three times and final pellet was dissolved in 30 μl RIPA buffer containing protease inhibitor cocktail. Lysates were freeze/thaw three times between −80C freezer and 37°C water bath by rigorously vortexing at each cycle. Final solutions were spun at 20.000g, 15 min at 4C. The supernatants were transferred into pre-chilled 1.5 mL tubes. 3 μl of each sample was used for protein assays. 10 μg of total lysate was loaded onto each well of a 10% polyacrylamide gel. Electrophoresis was performed at 100 V, 1 h 40’. Protein content was transferred onto polyvinylidene fluoride (PVDF) membrane at 0.5mA, 1h 40’. Membrane was stained with 0.5% Ponceau stain, individual blots were cut and processed with corresponding antibodies separately. Briefly, blots were blocked with blocking buffer [3% BSA in PBST (PBS, 0.5% Tween 20)]. Membranes were washed with PBST three times between the antibody incubations. Primary antibody incubations were performed using 1/1000 dilutions of the primary antibodies overnight at 4°C. Fluorescent secondary antibody incubations were performed at room temperature for 45 min. After the final wash blots were imaged using ChemiDoc image analyzer.

#### Transmission electron microscopy

At 24 h post-transfection, HEK293T or A549 cells were washed with Dulbecco’s phosphate buffered saline (PBS) and detached with 300 μL EDTA (10 mM) at 4°C for 10 min. The cell suspension was centrifuged at 350×g for 10 min at 4°C. Additionally, A549 cells were sorted in a CytoFlex SRT for those cells co transfected with GFP to enrich for transfected cells. EDTA was removed and the samples were re-suspended in 1mL of 2% paraformaldehyde and 2% glutaraldehyde in 0.1 M Cacodylate buffer for overnight fixation at 4C. Post fixation was done with 2% Osmium tetroxide for 2 h followed by 1% Tannic acid for 1h. Dehydration was followed by Spurrs embedding overnight. Sectioning was completed with glass knives and nickel mesh was used for placing thin sections (~70nm). After sectioning, 1% Uranyl Acetate for 10 min and lead citrate for 5 min were used to further stain membranes. Sections were then dried and kept away from moisture until imaging. Finished sections of HEK293T cells were imaged at magnifications on a Phillips transmission electron microscope, A549 cells were imaged on an FEI-Tecnai-12-Biotwin.

#### Cellular respirometry

Cellular respirometry was performed using the Agilent XFe24 Seahorse Bioanalyzer. Briefly, 2.0×105 HEK293T cells per well were seeded on poly-L-lysine coated seahorse plates and transfected 2 h later with 1 μg total DNA using Lipofectamine 2000. At 24 h post transfection, cell media was switch to unbuffered Seahorse DMEM medium pH 7.4 lacking NaHCO3 (Sigma) supplemented with 4.5 g/L D-glucose (Sigma), 1mM sodium pyruvate (Gibco), and 2% fatty acid free BSA (Sigma). A mitochondrial stress test was performed analyzing basal, ATP-uncoupled (17.3 mM oligomycin), and maximal (400 mM DNP) respirations. Finally, a cocktail of antimycin A (140 nM) and rotenone (7 mM) was used to abolish mitochondrial respiration to control for background non-mitochondrial respiration. Oxygen consumption rates (OCR) and extracellular acidification rates (ECAR) were assessed, with reagents specific for OCR reading, and normalized to total protein levels via BCA analysis (Pierce).

#### Microarray data processing

5-replicate wells for each time point for mock-infected and NiV-infected HEK293T cells were washed with 5 mL cold PBS and lysed with 1 mL of TRIzol (Invitrogen) according to the manufacturer’s recommendations. Extracted RNA was sent to Arraystar Inc. (Rockville, MD USA) for microarray analysis where it was hybridized to the Whole Human Genome Oligo Microarray (4 × 44K, Agilent Technologies). Arrays were scanned by the Agilent Scanner G2505C and raw images were analyzed using the Agilent Feature Extraction software to extract raw intensities, probe mappings, and quality-control (QC) metrics for each array. Extracted raw data were background corrected using the norm-exp method and quantile normalized using Bioconductor limma package, as previously described.^136,137^ Replicate probes were mean summarized into a single mRNA gene measure after passing Agilent QC flags.

### QUANTIFICATION AND STATISTICAL ANALYSIS

#### Data and statistical analyses

Peptide, metabolite and lipid features were subjected to statistical analysis for outliers, normalization and differential expression. The algorithm RMD-PAV^126^ was used to identify outlier biological samples, features with inadequate data for either qualitative or quantitative statistical tests were also removed from the dataset, and SPANS was used^127^ for normalization. Peptide, transcript, lipid, and metabolite features were transformed to log_2_ fold-changes (FC) over their time-matched mock controls and assessed for statistical significance by ANOVA (quantitative) or g-test (qualitative), where applicable. From transcriptomics and proteomics, only transcripts and proteins of human origin were considered and in the case of proteomics, only genes with enough analyzed peptides for quantitative assessment (via ANOVA) of fold-change were considered. Furthermore, FC and *p-value* cut-offs were set as log_2_ FC ≤ −0.58 or ≥0.58 (equivalent to ±1.5-fold change) and *p* ≤ 0.05 for transcriptomics and proteomics. In order to sub-categorize the list of the proteins coming from infection and transfection experiments, we used the trend values. To compare proteins, we refined the raw data using two criteria; upregulation compared to mock by statistical significance and peptide abundance ≥4. The final lists were run on Cytoscape and sub-categorized using search key terms function. Sub-categorized lists from infection and transfection experiments were compared. For metabolomics and lipidomics, significance was based on ANOVA (*p* ≤ 0.05). After identifying proteins and genes that were significantly altered in abundance (based on peptide peaks, see proteomics section) during infection, the gene ontology (GO) software, GOTERMFINDER,^128^ and the GO redundancy removal software, REVIGO,^56^ were used together to analyze datasets by identifying cellular pathways most associated with the affected proteins. InteractiVenn^129^ was used to compare between gene/protein or GO cellular process lists between time-points, as necessary. For REVIGO, medium stringency (redundancy score <0.7) was used for both proteomics and transcriptomics. For construction of the heatmap used for transcriptomics, the software Heatmapper was used.^130^

Due to the quantity of data produced from the proteomics experiments, another tool, Reactome,^57,131^ was used to more easily visualize which cellular processes were affected during infection based on the abundance changes of proteins associated with each highlighted cellular process. The dataset used for this analysis included all proteins meeting both statistical and FC cut-offs (as discussed above) for at least one time-point. For each protein, log_2_ FC values were given for each time-point and blank values were substituted with 0 (no fold difference over mock) as required for Reactome to run. Table S2 includes the data in this format to support future reader analysis with the Reactome software.

#### Proteomics, lipidomics, metabolomics

Statistical analyses were performed separately for proteomics, lipidomics, and metabolomics data, using the quantified biomolecule peak intensities. For each data type, values that were not observed were indicated by NA and data were then log2 transformed. Biomolecules not observed in at least two samples across all instrument runs within a study were removed. RMD-PAV^126^ (*p*-value threshold 0.001) and Pearson correlation were used to identify samples that were biological outliers. The lipid and metabolite datasets were normalized using global median centering. SPANS^127^ was used to identify an appropriate normalization strategy for the peptide data, selecting global median centering. Peptides, lipids, and metabolites were evaluated by Analysis of Variance (ANOVA) with a Dunnett test correction and a Bonferroni-corrected g-test^132^ to compare Nipah virus samples to the mock samples within each time point (4, 8, 12, and 16 h post-infection). BP-Quant^133^ (default parameter 0.9) was used to perform signature-based protein quantification, where each peptide was categorized as 0, 1, or −1 according to the following rules where significance was defined by an adjusted *p*-value <0.05. Peptides significant by ANOVA with a negative virus-to-mock fold change or significant by g-test with fewer observed values in virus samples than mock samples were categorized as −1; peptides significant by ANOVA with a positive virus-to-mock fold change or significant by g-test with more observed values in virus samples than mock samples were categorized as 1; peptides not significant by either test were categorized as 0. After protein quantification, all proteins were analyzed using the same methodology as for the other biomolecules: ANOVA with a Dunnett test correction and a Bonferroni-corrected g-test to compare Nipah virus samples to mock samples within each time point.

## Supplementary Material

1

2

3

4

5

## Figures and Tables

**KEY RESOURCES TABLE:** 

REAGENT or RESOURCE	SOURCE	IDENTIFIER

Antibodies		

Mouse monoclonal anti-Myc	ThermoFisher	Cat# MA-1-21316; RRID:AB_2536992
Rabbit monoclonal anti-T7	Cell Signaling	Cat# 13246S
Mouse monoclonal anti-6xH	Biolegend	Cat# 652505; RRID:AB_2564271
Mouse monoclonal anti-AU5	Biolegend	Cat# 902002; RRID:AB_2565015
Rabbit polyclonal anti-E	ThermoFisher	Cat# PA5-81630; RRID:AB_2788823
Mouse monoclonal anti-Flag	Krackler Scientific	Cat# 45-F3165
Mouse monoclonal anti-AU1	Biolegend	Cat# 901902; RRID:AB_2565014
Mouse monoclonal anti-HA	Biolegend	Cat# 901503; RRID:AB_2565005

Bacterial and virus strains		

DH5a	ThermoFisher	Cat# DH5a
NiV	NIH	Malaysia strain

Chemicals, peptides, and recombinant proteins		

MitoTracker^®^ FM	ThermoFisher	Cat# M7514
HR2 peptide	Aguilar et al.^123^ Zamora et al.^124,125^	KVDISSQISSMNQSLQQSKDYI KEAQRLLDTVNPSL
ethylenediamine tetraacetic acid (ETDA)	VWR Chemicals	Cat# VWRC20294.294
Chloroform	VWR Chemicals	Cat# VWRC84111.0100
Methanol	VWR Chemicals	Cat# VWRC0323-4L
media lb-agar samp capsule sterile 454 g	VWR International	Cat# 76019-958
tris(carboxyethyl) phosphine	Krackeler	Cat# 45-C4706-2G-EA
Magnesium chloride, anhydrous 99%	VWR International	Cat# ALFA12315.A1
Benzonase^®^ Nuclease	MiliporeSigma	Cat# E1014-25KU
dithiothreitol	MiliporeSigma	Cat# 43815-5G
iodoacetamide	MiliporeSigma	Cat# I1149-25G
ammonium bicarbonate	MiliporeSigma	Cat# A6141-25G
CaCl_2_	MiliporeSigma	Cat# C5670-500G
Trypsin-EDTA (0.25%), phenol red	Life Technology	Cat# 25200072
acetonitrile	MiliporeSigma	Cat# 34851-2.5L
trifluoroacetic acid	MiliporeSigma	Cat# 302031-100ML
formic acid	MiliporeSigma	Cat# 33015-500ML
silica	MiliporeSigma	Cat# 381276-500G
methoxyamine	MiliporeSigma	Cat# 226904-25G
pyridine	MiliporeSigma	Cat# 360570-500ML
trifluoroacetamide	MiliporeSigma	Cat# M7891-5G
trimethylchlorosilane	MiliporeSigma	Cat# 386529-100ML
ammonium acetate	MiliporeSigma	Cat# A1542-500G
ethanol	MiliporeSigma	Cat# 459844-1L
thioanisole	MiliporeSigma	Cat# T28002-100G
polyvinylidene fluoride	MiliporeSigma	Cat# 1155460001
polyethylene-imine	MiliporeSigma	Cat# 408727-250ML
bovine serum albumin	VWR International	Cat# 45000-734
phosphate-buffered saline/500mL	VWR International	Cat# 45000-434
Tween 20	MiliporeSigma	Cat# 655204-100ML
cacodylate	MiliporeSigma	Cat# C4945-25G
osmium tetroxide	MiliporeSigma	Cat# 201030-250MG
tannic acid	MiliporeSigma	Cat# 1007731000
uranyl acetate. (Uranyl acetate 3% in aqueous 100 mL solution)	VWR International	Cat# 102092-286
poly-L-lysine	MiliporeSigma	Cat# 45-P4707-50ML-EA
D-glucose	MiliporeSigma	Cat# 1370481000
sodium pyruvate	ThermoFisher	Cat# J61840.22
oligomycin – 100mg	ThermoFisher	Cat# J61898.MA
antimycin A	Sigma Aldrich	Cat# A8674-25MG
rotenone	Sigma Aldrich	Cat# 557368-1GM
TRIzol (Invitrogen)	ThermoFisher	Cat# 15596018
Dulbecco's Modified Eagle Medium (Gibco) (Corning)	VWR International	Cat# 10-016-CM
fetal bovine serum (Gibco) *(We buy ATLAS scientific)	Atlas Bilogical	Cat# F-0500-D

Critical commercial assays		

SpeeDNA Isolation Kit	ScienCell	Cat# MB6918
Absolute Human Mitochondrial DNA Copy	ScienCell	Cat# 8948
Number Quantification qPCR Assay Kit

Deposited data		

NiV infection proteomics	PRIDE; This paper	MSV000086754
NiV infection transcriptomics	ArrayExpress; This paper	GSE166707
NiV infection metabolomics	Metabolights; This paper	MSV000086735
NiV infection lipidomics	BioSamples; This paper	MSV000086736
NiV F and NiV G transfection proteomics	PRIDE; This paper	MSV000086754
NiV F and NiV G transfection transcriptomics	ArrayExpress; This paper	GSE166698, GSE166699, GSE166700, GSE166701, GSE166702, GSE166706
NiV F and NiV G transfection metabolomics	Metabolights; This paper	MSV000086735
NiV F and NiV G transfection lipidomics	BioSamples; This paper	MSV000086736

Experimental models: Cell lines		

Human: HEK293T	ATCC	CRL-3216
Human: A549	ATCC	CCL-185

Recombinant DNA		

Plasmid: NiV N	This paper, See Table S6	N/A
Plasmid: NiV P	This paper, See Table S6	N/A
Plasmid: NiV W	This paper, See Table S6	N/A
Plasmid: NiV N	This paper, See Table S6	N/A
Plasmid: NiV V	This paper, See Table S6	N/A
Plasmid: NiV M	Johnston et al.^47^ See Table S6	N/A
Plasmid: NiV F	Aguilar et al.^134^ See Table S6	N/A
Plasmid: NiV G	Aguilar et al.^134^ See Table S6	N/A
Plasmid: NiV L	This paper, See Table S6	N/A
Plasmid: RFP-HA	Addgene	Cat# 90265
Plasmid: pcDNA3.1 +	Addgene	Cat# V790-20

Software and algorithms		

Cytoscape (STRING plug in)	Web	https://cytoscape.org/
Gene Ontology	web	http://geneontology.org/
Revigo	web	http://revigo.irb.hr/
Reactome	web	https://reactome.org/
Lipid Mini-On	web	https://omicstools.pnnl.gov/shiny/lipid-mini-on/
gotermfinder	web	https://go.princeton.edu/cgi-bin/GOTermFinder
Heatmapper	web	http://www.heatmapper.ca/
interactivenn	web	http://www.interactivenn.net/
Fiji Software	Schindelin et al.^135^	https://fiji.sc/

Other		

Polyethylene-imine	Polysciences	Cat# 23966
Lipofectamine 2000	ThermoFisher	Cat# 11668019
Lipofectamine 3000	ThermoFisher	Cat# L3000008

## Highlights

- This study identified cellular changes upon human cell infection by the deadly Nipah virus
- We used a multi-platform transcriptomics, proteomics, lipidomics, and metabolomics approach
- This study implicated mitochondria-associated protein enrichment and metabolic changes
- These analyses provide an atlas of cellular changes that may help in designing therapeutics

## References

[1] Chua KB, Bellini WJ, Rota PA, Harcourt BH, Tamin A, Lam SK, Ksiazek TG, Rollin PE, Zaki SR, Shieh W, (2000). Nipah Virus: A Recently Emergent Deadly Paramyxovirus. Science 288, 1432–1435. 10.1126/science.288.5470.1432. 10827955

[2] Ching PKG, de los Reyes VC, Sucaldito MN, Tayag E, Columna-Vingno AB, Malbas FF, Bolo GC, Sejvar JJ, Eagles D, Playford G, (2015). Outbreak of Henipavirus Infection, Philippines, 2014. Emerg. Infect. Dis. 21, 328–331. 10.3201/eid2102.141433. 25626011 PMC4313660

[3] Lam SK, and Chua KB (2002). Nipah Virus Encephalitis Outbreak in Malaysia. Clin. Infect. Dis. 34, S48–S51. 10.1086/338818. 11938496

[4] Luby SP, Hossain MJ, Gurley ES, Ahmed B-N, Banu S, Khan SU, Homaira N, Rota PA, Rollin PE, Comer JA, (2009). Recurrent Zoonotic Transmission of Nipah Virus into Humans, Bangladesh, 2001–2007. Emerg. Infect. Dis. 15, 1229–1235. 10.3201/eid1508.081237. 19751584 PMC2815955

[5] Nipah Virus Outbreaks in the WHO South-East Asia Region (World Health Organization).

[6] Drexler JF, Corman VM, Müller MA, Maganga GD, Vallo P, Binger T, Gloza-Rausch F, Cottontail VM, Rasche A, Yordanov S, (2012). Bats host major mammalian paramyxoviruses. Nat. Commun. 3, 796. 10.1038/ncomms1796. 22531181 PMC3343228

[7] Wu Z, Yang L, Yang F, Ren X, Jiang J, Dong J, Sun L, Zhu Y, Zhou H, and Jin Q (2014). Novel Henipa-like Virus, Mojiang Paramyxovirus, in Rats, China, 2012. Emerg. Infect. Dis. 20, 1064–1066. 10.3201/eid2006.131022. 24865545 PMC4036791

[8] Marsh GA, de Jong C, Barr JA, Tachedjian M, Smith C, Middleton D, Yu M, Todd S, Foord AJ, Haring V, (2012). Cedar Virus: A Novel Henipavirus Isolated from Australian Bats. PLoS Pathog. 8, e1002836. 10.1371/journal.ppat.1002836. 22879820 PMC3410871

[9] Pernet O, Schneider BS, Beaty SM, LeBreton M, Yun TE, Park A, Zachariah TT, Bowden TA, Hitchens P, Ramirez CM, (2014). Evidence for henipavirus spillover into human populations in Africa. Nat. Commun. 5, 5342. 10.1038/ncomms6342. 25405640 PMC4237230

[10] Breed AC, Meers J, Sendow I, Bossart KN, Barr JA, Smith I, Wacharapluesadee S, Wang L, and Field HE (2013). The Distribution of Henipaviruses in Southeast Asia and Australasia: Is Wallace’s Line a Barrier to Nipah Virus? PLoS One 8, e61316. 10.1371/journal.pone.0061316. 23637812 PMC3634832

[11] Curran J, and Kolakofsky D (1999). Replication of paramyxoviruses. Adv. Virus Res. 54, 403–422. 10547681 10.1016/s0065-3527(08)60373-5

[12] Ranadheera C, Proulx R, Chaiyakul M, Jones S, Grolla A, Leung A, Rutherford J, Kobasa D, Carpenter M, and Czub M (2018). The interaction between the Nipah virus nucleocapsid protein and phosphoprotein regulates virus replication. Sci. Rep. 8, 15994. 10.1038/s41598-018-34484-7. 30375468 PMC6207681

[13] Halpin K, Bankamp B, Harcourt BH, Bellini WJ, and Rota PA (2004). Nipah virus conforms to the rule of six in a minigenome replication assay. J. Gen. Virol. 85, 701–707. 10.1099/vir.0.19685-0. 14993656

[14] Wong KT, Shieh W-J, Kumar S, Norain K, Abdullah W, Guarner J, Goldsmith CS, Chua KB, Lam SK, Tan CT, (2002). Nipah virus infection: pathology and pathogenesis of an emerging paramyxoviral zoonosis. Am. J. Pathol. 161, 2153–2167. 10.1016/S0002-9440(10)64493-8. 12466131 PMC1850894

[15] Geisbert TW, Daddario-DiCaprio KM, Hickey AC, Smith MA, Chan Y-P, Wang L-F, Mattapallil JJ, Geisbert JB, Bossart KN, and Broder CC (2010). Development of an Acute and Highly Pathogenic Nonhuman Primate Model of Nipah Virus Infection. PLoS One 5, e10690. 10.1371/journal.pone.0010690. 20502528 PMC2872660

[16] Baseler L, Scott DP, Saturday G, Horne E, Rosenke R, Thomas T, Meade-White K, Haddock E, Feldmann H, and de Wit E (2016). Identifying Early Target Cells of Nipah Virus Infection in Syrian Hamsters. PLoS Negl. Trop. Dis. 10, e0005120. 10.1371/journal.pntd.0005120. 27812087 PMC5094696

[17] Escaffre O, Borisevich V, and Rockx B (2013). Pathogenesis of Hendra and Nipah virus infection in humans. J. Infect. Dev. Ctries. 7, 308–311. 10.3855/jidc.3648. 23592639

[18] DeBuysscher BL, de Wit E, Munster VJ, Scott D, Feldmann H, and Prescott J (2013). Comparison of the Pathogenicity of Nipah Virus Isolates from Bangladesh and Malaysia in the Syrian Hamster. PLoS Negl. Trop. Dis. 7, e2024. 10.1371/journal.pntd.0002024. 23342177 PMC3547834

[19] Mungall BA, Middleton D, Crameri G, Bingham J, Halpin K, Russell G, Green D, McEachern J, Pritchard LI, Eaton BT, (2006). Feline Model of Acute Nipah Virus Infection and Protection with a Soluble Glycoprotein-Based Subunit Vaccine. J. Virol. 80, 12293–12302. 10.1128/JVI.01619-06. 17005664 PMC1676295

[20] Mills JN, Alim ANM, Bunning ML, Lee OB, Wagoner KD, Amman BR, Stockton PC, and Ksiazek TG (2009). Nipah Virus Infection in Dogs, Malaysia, 1999. Emerg. Infect. Dis. 15, 950–952. 10.3201/eid1506.080453. 19523300 PMC2727347

[21] Looi L-M, and Chua K-B (2007). Lessons from the Nipah virus outbreak in Malaysia. Malays. J. Pathol. 29, 63–67. 19108397

[22] Islam MS, Sazzad HMS, Satter SM, Sultana S, Hossain MJ, Hasan M, Rahman M, Campbell S, Cannon DL, Ströher U, (2016). Nipah Virus Transmission from Bats to Humans Associated with Drinking Traditional Liquor Made from Date Palm Sap, Bangladesh, 2011–2014. Emerg. Infect. Dis. 22, 664–670. 10.3201/eid2204.151747. 26981928 PMC4806957

[23] Gurley ES, Hegde ST, Hossain K, Sazzad HMS, Hossain MJ, Rahman M, Sharker MAY, Salje H, Islam MS, Epstein JH, (2017). Convergence of Humans, Bats, Trees, and Culture in Nipah Virus Transmission, Bangladesh. Emerg. Infect. Dis. 23, 1446–1453. 10.3201/eid2309.161922. 28820130 PMC5572889

[24] Paul L (2018). Nipah virus in Kerala : A deadly Zoonosis. Clin. Microbiol. Infect. 24, 1113–1114. 10.1016/j.cmi.2018.06.017. 29935330

[25] Chang L-Y, Ali ARM, Hassan SS, and AbuBakar S (2007). Human neuronal cell protein responses to Nipah virus infection. Virol. J. 4, 54. 10.1186/1743-422X-4-54. 17553172 PMC1896155

[26] Escaffre O, Borisevich V, Carmical JR, Prusak D, Prescott J, Feldmann H, and Rockx B (2013). Henipavirus Pathogenesis in Human Respiratory Epithelial Cells. J. Virol. 87, 3284–3294. 10.1128/JVI.02576-12. 23302882 PMC3592112

[27] de Wit E, and Munster VJ (2015). Animal models of disease shed light on Nipah virus pathogenesis and transmission: Nipah virus pathogenesis and transmission. J. Pathol. 235, 196–205. 10.1002/path.4444. 25229234 PMC4268059

[28] Munster VJ, Prescott JB, Bushmaker T, Long D, Rosenke R, Thomas T, Scott D, Fischer ER, Feldmann H, and de Wit E (2012). Rapid Nipah virus entry into the central nervous system of hamsters via the olfactory route. Sci. Rep. 2, 736. 10.1038/srep00736. 23071900 PMC3471094

[29] Basler CF (2012). Nipah and Hendra Virus Interactions with the Innate Immune System. In Henipavirus B Lee and Rota PA, eds. (Springer Berlin Heidelberg), pp. 123–152. 10.1007/82_2012_209. 22491899

[30] Prescott J, de Wit E, Feldmann H, and Munster VJ (2012). The immune response to Nipah virus infection. Arch. Virol. 157, 1635–1641. 10.1007/s00705-012-1352-5. 22669317 PMC3432143

[31] Seto J, Qiao L, Guenzel CA, Xiao S, Shaw ML, Hayot F, and Sealfon SC (2010). Novel Nipah Virus Immune-Antagonism Strategy Revealed by Experimental and Computational Study. J. Virol. 84, 10965–10973. 10.1128/JVI.01335-10. 20739535 PMC2953155

[32] Sánchez-Aparicio MT, Feinman LJ, García-Sastre A, and Shaw ML (2018). Paramyxovirus V Proteins Interact with the RIG-I/TRIM25 Regulatory Complex and Inhibit RIG-I Signaling. J. Virol. 92, e01960–17. 10.1128/JVI.01960-17. 29321315 PMC5827389

[33] Bharaj P, Wang YE, Dawes BE, Yun TE, Park A, Yen B, Basler CF, Freiberg AN, Lee B, and Rajsbaum R (2016). The Matrix Protein of Nipah Virus Targets the E3-Ubiquitin Ligase TRIM6 to Inhibit the IKKε Kinase-Mediated Type-I IFN Antiviral Response. PLoS Pathog. 12, e1005880. 10.1371/journal.ppat.1005880. 27622505 PMC5021333

[34] Chan EY, Qian W-J, Diamond DL, Liu T, Gritsenko MA, Monroe ME, Camp DG, Smith RD, and Katze MG (2007). Quantitative Analysis of Human Immunodeficiency Virus Type 1-Infected CD4+ Cell Proteome: Dysregulated Cell Cycle Progression and Nuclear Transport Coincide with Robust Virus Production. J. Virol. 81, 7571–7583. 10.1128/JVI.00288-07. 17494070 PMC1933372

[35] Hu A, Noble WS, and Wolf-Yadlin A (2016). Technical advances in proteomics: new developments in data-independent acquisition. F1000Res. 5, F1000–Faculty. 10.12688/f1000research.7042.1. PMC4821292 27092249

[36] Kao A, Chiu C.l., Vellucci D, Yang Y, Patel VR, Guan S, Randall A, Baldi P, Rychnovsky SD, and Huang L (2011). Development of a Novel Cross-linking Strategy for Fast and Accurate Identification of Cross-linked Peptides of Protein Complexes. Mol. Cell. Proteomics 10, M110.002212. 10.1074/mcp.M110.002212. PMC3013449 20736410

[37] Jiang X-S, Tang L-Y, Dai J, Zhou H, Li S-J, Xia Q-C, Wu J-R, and Zeng R (2005). Quantitative Analysis of Severe Acute Respiratory Syndrome (SARS)-associated Coronavirus-infected Cells Using Proteomic Approaches: Implications for Cellular Responses to Virus Infection. Mol. Cell. Proteomics 4, 902–913. 10.1074/mcp.M400112-MCP200. 15784933 PMC7780044

[38] Kaake RM, Wang X, Burke A, Yu C, Kandur W, Yang Y, Novtisky EJ, Second T, Duan J, Kao A, (2014). A New *in Vivo* Cross-linking Mass Spectrometry Platform to Define Protein–Protein Interactions in Living Cells. Mol. Cell. Proteomics 13, 3533–3543. 10.1074/mcp.M114.042630. 25253489 PMC4256503

[39] Munday DC, Emmott E, Surtees R, Lardeau C-H, Wu W, Duprex WP, Dove BK, Barr JN, and Hiscox JA (2010). Quantitative Proteomic Analysis of A549 Cells Infected with Human Respiratory Syncytial Virus. Mol. Cell. Proteomics 9, 2438–2459. 10.1074/mcp.M110.001859. 20647383 PMC2984239

[40] Vidova V, and Spacil Z (2017). A review on mass spectrometry-based quantitative proteomics: Targeted and data independent acquisition. Anal. Chim. Acta 964, 7–23. 10.1016/j.aca.2017.01.059. 28351641

[41] Oxford KL, Wendler JP, McDermott JE, White Iii RA, Powell JD, Jacobs JM, Adkins JN, and Waters KM (2016). The landscape of viral proteomics and its potential to impact human health. Expert Rev. Proteomics 13, 579–591. 10.1080/14789450.2016.1184091. 27133506

[42] Vera-Velasco NM, García-Murria MJ, Sánchez del Pino MM, Mingarro I, and Martinez-Gil L (2018). Proteomic composition of Nipah virus -like particles. J. Proteonomics 172, 190–200. 10.1016/j.jprot.2017.10.012. 29092793

[43] Martinez-Gil L, Vera-Velasco NM, and Mingarro I (2017). Exploring the Human-Nipah Virus Protein-Protein Interactome. J. Virol. 91, e01461–17. 10.1128/JVI.01461-17. 28904190 PMC5686741

[44] Liu Q, Stone JA, Bradel-Tretheway B, Dabundo J, Benavides Montano JA, Santos-Montanez J, Biering SB, Nicola AV, Iorio RM, Lu X, and Aguilar HC (2013). Unraveling a Three-Step Spatiotemporal Mechanism of Triggering of Receptor-Induced Nipah Virus Fusion and Cell Entry. PLoS Pathog. 9, e1003770. 10.1371/journal.ppat.1003770. 24278018 PMC3837712

[45] Wynne JW, Shiell BJ, Marsh GA, Boyd V, Harper JA, Heesom K, Monaghan P, Zhou P, Payne J, Klein R, (2014). Proteomics informed by transcriptomics reveals Hendra virus sensitizes bat cells to TRAIL mediated apoptosis. Genome Biol. 15, 532. 10.1186/PREACCEPT-1718798964145132. 25398248 PMC4269970

[46] Aguilar HC, Matreyek KA, Choi DY, Filone CM, Young S, and Lee B (2007). Polybasic KKR Motif in the Cytoplasmic Tail of Nipah Virus Fusion Protein Modulates Membrane Fusion by Inside-Out Signaling. J. Virol. 81, 4520–4532. 10.1128/JVI.02205-06. 17301148 PMC1900187

[47] Johnston GP, Contreras EM, Dabundo J, Henderson BA, Matz KM, Ortega V, Ramirez A, Park A, and Aguilar HC (2017). Cytoplasmic Motifs in the Nipah Virus Fusion Protein Modulate Virus Particle Assembly and Egress. J. Virol. 91, 10–1128. 10.1128/JVI.02150-16. PMC5411590 28250132

[48] Cifuentes-Muñoz N, Sun W, Ray G, Schmitt PT, Webb S, Gibson K, Dutch RE, and Schmitt AP (2017). Mutations in the Transmembrane Domain and Cytoplasmic Tail of Hendra Virus Fusion Protein Disrupt Virus-Like-Particle Assembly. J. Virol. 91, 10–1128. 10.1128/JVI.00152-17. PMC5487568 28468881

[49] Burnum-Johnson KE, Kyle JE, Eisfeld AJ, Casey CP, Stratton KG, Gonzalez JF, Habyarimana F, Negretti NM, Sims AC, Chauhan S, (2017). MPLEx: a method for simultaneous pathogen inactivation and extraction of samples for multi-omics profiling. Analyst 142, 442–448. 10.1039/c6an02486f. 28091625 PMC5283721

[50] Duy J, Koehler JW, Honko AN, and Minogue TD (2015). Optimized microRNA purification from TRIzol-treated plasma. BMC Genom. 16, 95. 10.1186/s12864-015-1299-5. PMC4342875 25765146

[51] Shurtleff AC, Garza N, Lackemeyer M, Carrion R, Griffiths A, Patterson J, Edwin SS, and Bavari S (2012). The Impact of Regulations, Safety Considerations and Physical Limitations on Research Progress at Maximum Biocontainment. Viruses 4, 3932–3951. 10.3390/v4123932. 23342380 PMC3528297

[52] Blow JA, Dohm DJ, Negley DL, and Mores CN (2004). Virus inactivation by nucleic acid extraction reagents. J. Virol. Methods 119, 195–198. 10.1016/j.jviromet.2004.03.015. 15158603

[53] Feldmann F, Shupert WL, Haddock E, Twardoski B, and Feldmann H (2019). Gamma Irradiation as an Effective Method for Inactivation of Emerging Viral Pathogens. Am. J. Trop. Med. Hyg. 100, 1275–1277. 10.4269/ajtmh.18-0937. 30860018 PMC6493948

[54] Haddock E, Feldmann F, and Feldmann H (2016). Effective Chemical Inactivation of Ebola Virus. Emerg. Infect. Dis. 22, 1292–1294. 10.3201/eid2207.160233. 27070504 PMC4918181

[55] Blumberg BM, Chan J, and Udem SA (1991). Function of Paramyxo-virus 3′ and 5′ End Sequences. In The Paramyxoviruses the Viruses, Kingsbury DW, ed. (Springer US), pp. 235–247. 10.1007/978-1-4615-3790-8_8.

[56] Supek F, Bošnjak M, Škunca N, and Šmuc T (2011). REVIGO Summarizes and Visualizes Long Lists of Gene Ontology Terms. PLoS One 6, e21800. 10.1371/journal.pone.0021800. 21789182 PMC3138752

[57] Haw R, Hermjakob H, D’Eustachio P, and Stein L (2011). Reactome pathway analysis to enrich biological discovery in proteomics data sets. Proteomics 11, 3598–3613. 10.1002/pmic.201100066. 21751369 PMC4617659

[58] Rojas M, Arias CF, and Lopez S (2010). Protein Kinase R Is Responsible for the Phosphorylation of eIF2α in Rotavirus Infection. J. Virol. 84, 10457–10466. 10.1128/JVI.00625-10. 20631127 PMC2950594

[59] Sadler AJ, and Williams BRG (2007). Structure and function of the protein kinase R. Curr. Top. Microbiol. Immunol. 316, 253–292. 17969452 10.1007/978-3-540-71329-6_13

[60] Habjan M, Pichlmair A, Elliott RM, Overby AK, Glatter T, Gstaiger M, Superti-Furga G, Unger H, and Weber F (2009). NSs Protein of Rift Valley Fever Virus Induces the Specific Degradation of the Double-Stranded RNA-Dependent Protein Kinase. J. Virol. 83, 4365–4375. 10.1128/JVI.02148-08. 19211744 PMC2668506

[61] Ikegami T, Narayanan K, Won S, Kamitani W, Peters CJ, and Makino S (2009). Rift Valley Fever Virus NSs Protein Promotes Post-Transcriptional Downregulation of Protein Kinase PKR and Inhibits eIF2α Phosphorylation. PLoS Pathog. 5, e1000287. 10.1371/journal.ppat.1000287. 19197350 PMC2629125

[62] Hajnóczky G, Csordás G, Das S, Garcia-Perez C, Saotome M, Sinha Roy S, and Yi M (2006). Mitochondrial calcium signalling and cell death: Approaches for assessing the role of mitochondrial Ca2+ uptake in apoptosis. Cell Calcium 40, 553–560. 10.1016/j.ceca.2006.08.016. 17074387 PMC2692319

[63] McBride HM, Neuspiel M, and Wasiak S (2006). Mitochondria: MoreThan Just a Powerhouse. Curr. Biol. 16, R551–R560. 10.1016/j.cub.2006.06.054. 16860735

[64] Anand SK, and Tikoo SK (2013). Viruses as Modulators of Mitochondrial Functions. Adv. Virol. 2013, 738794–738817. 10.1155/2013/738794. 24260034 PMC3821892

[65] Khan M, Syed GH, Kim S-J, and Siddiqui A (2015). Mitochondrial dynamics and viral infections: A close nexus. Biochim. Biophys. Acta 1853, 2822–2833. 10.1016/j.bbamcr.2014.12.040. 25595529 PMC4500740

[66] Clair G, Reehl S, Stratton KG, Monroe ME, Tfaily MM, Ansong C, and Kyle JE (2019). Lipid Mini-On: mining and ontology tool for enrichment analysis of lipidomic data. Bioinformatics 35, 4507–4508. 10.1093/bioinformatics/btz250. 30977807 PMC7963073

[67] Tanner LB, Chng C, Guan XL, Lei Z, Rozen SG, and Wenk MR (2014). Lipidomics identifies a requirement for peroxisomal function during influenza virus replication. J. Lipid Res. 55, 1357–1365. 10.1194/jlr.M049148. 24868094 PMC4076098

[68] Waheed AA, and Freed EO (2010). The Role of Lipids in Retrovirus Replication. Viruses 2, 1146–1180. 10.3390/v2051146. 20740061 PMC2927015

[69] Kyle JE, Burnum-Johnson KE, Wendler JP, Eisfeld AJ, Halfmann PJ, Watanabe T, Sahr F, Smith RD, Kawaoka Y, Waters KM, and Metz TO (2019). Plasma lipidome reveals critical illness and recovery from human Ebola virus disease. Proc. Natl. Acad. Sci. USA 116, 3919–3928. 10.1073/pnas.1815356116. 30808769 PMC6397561

[70] Eisfeld AJ, Halfmann PJ, Wendler JP, Kyle JE, Burnum-Johnson KE, Peralta Z, Maemura T, Walters KB, Watanabe T, Fukuyama S, (2017). Multi-platform ‘Omics Analysis of Human Ebola Virus Disease Pathogenesis. Cell Host Microbe 22, 817–829.e8. 10.1016/j.chom.2017.10.011. 29154144 PMC5730472

[71] Vasaikar S, Huang C, Wang X, Petyuk VA, Savage SR, Wen B, Dou Y, Zhang Y, Shi Z, Arshad OA, (2019). Proteogenomic Analysis of Human Colon Cancer Reveals New Therapeutic Opportunities. Cell 177, 1035–1049.e19. 10.1016/j.cell.2019.03.030. 31031003 PMC6768830

[72] Zhang H, Liu T, Zhang Z, Payne SH, Zhang B, McDermott JE, Zhou J-Y, Petyuk VA, Chen L, Ray D, (2016). Integrated proteogenomic characterization of human high grade serous ovarian cancer. Cell 166, 755–765. 10.1016/j.cell.2016.05.069. 27372738 PMC4967013

[73] Kloetzel P-M (2004). The proteasome and MHC class I antigen processing. Biochim. Biophys. Acta 1695, 225–233. 10.1016/j.bbamcr.2004.10.004. 15571818

[74] Sijts EJAM, and Kloetzel P-M (2011). The role of the proteasome in the generation of MHC class I ligands and immune responses. Cell. Mol. Life Sci. 68, 1491–1502. 10.1007/s00018-011-0657-y. 21387144 PMC3071949

[75] Shannon P, Markiel A, Ozier O, Baliga NS, Wang JT, Ramage D, Amin N, Schwikowski B, and Ideker T (2003). Cytoscape: a software environment for integrated models of biomolecular interaction networks. Genome Res. 13, 2498–2504. 10.1101/gr.1239303. 14597658 PMC403769

[76] Cox J, and Mann M (2011). Quantitative, High-Resolution Proteomics for Data-Driven Systems Biology. Annu. Rev. Biochem. 80, 273–299. 10.1146/annurev-biochem-061308-093216. 21548781

[77] Wu H, Carvalho P, and Voeltz GK (2018). Here, there, and everywhere: The importance of ER membrane contact sites. Science 361, eaan5835. 10.1126/science.aan5835. 30072511 PMC6568312

[78] Chang L-Y, Ali ARM, Hassan SS, and AbuBakar S (2006). Nipah virus RNA synthesis in cultured pig and human cells. J. Med. Virol. 78, 1105–1112. 10.1002/jmv.20669. 16789019

[79] Valm AM, Cohen S, Legant WR, Melunis J, Hershberg U, Wait E, Cohen AR, Davidson MW, Betzig E, and Lippincott-Schwartz J (2017). Applying systems-level spectral imaging and analysis to reveal the organelle interactome. Nature 546, 162–167. 10.1038/nature22369. 28538724 PMC5536967

[80] Prinz WA, Toulmay A, and Balla T (2020). The functional universe of membrane contact sites. Nat. Rev. Mol. Cell Biol. 21, 7–24. 10.1038/s41580-019-0180-9. 31732717 PMC10619483

[81] Pfanner N, Warscheid B, and Wiedemann N (2019). Mitochondrial proteins: from biogenesis to functional networks. Nat. Rev. Mol. Cell Biol. 20, 267–284. 10.1038/s41580-018-0092-0. 30626975 PMC6684368

[82] Vance JE (1990). Phospholipid synthesis in a membrane fraction associated with mitochondria. J. Biol. Chem. 265, 7248–7256. 2332429

[83] Phielix E, Schrauwen-Hinderling VB, Mensink M, Lenaers E, Meex R, Hoeks J, Kooi ME, Moonen-Kornips E, Sels J-P, Hesselink MKC, and Schrauwen P (2008). Lower intrinsic ADP-stimulated mitochondrial respiration underlies in vivo mitochondrial dysfunction in muscle of male type 2 diabetic patients. Diabetes 57, 2943–2949. 10.2337/db08-0391. 18678616 PMC2570390

[84] Lee HC, Yin PH, Lu CY, Chi CW, and Wei YH (2000). Increase of mitochondria and mitochondrial DNA in response to oxidative stress in human cells. Biochem. J. 348 Pt 2, 425–432. 10816438 PMC1221082

[85] Twig G, Hyde B, and Shirihai OS (2008). Mitochondrial fusion, fission and autophagy as a quality control axis: The bioenergetic view. Biochim. Biophys. Acta 1777, 1092–1097. 10.1016/j.bbabio.2008.05.001. 18519024 PMC3809017

[86] Haimovitz-Friedman A, Kolesnick RN, and Fuks Z (1997). Ceramide signaling in apoptosis. Br. Med. Bull. 53, 539–553. 10.1093/OXFORDJOURNALS.BMB.A011629. 9374036

[87] Kvansakul M (2017). Viral Infection and Apoptosis. Viruses 9, 356. 10.3390/v9120356. 29168732 PMC5744131

[88] Okamoto T, Suzuki T, Kusakabe S, Tokunaga M, Hirano J, Miyata Y, and Matsuura Y (2017). Regulation of Apoptosis during Flavivirus Infection. Viruses 9, 243. 10.3390/v9090243. 28846635 PMC5618009

[89] Shim JM, Kim J, Tenson T, Min J-Y, and Kainov DE (2017). Influenza Virus Infection, Interferon Response, Viral Counter-Response, and Apoptosis. Viruses 9, 223. 10.3390/v9080223. 28805681 PMC5580480

[90] Dykxhoorn DM, and Lieberman J (2006). Silencing Viral Infection. PLoS Med. 3, e242. 10.1371/journal.pmed.0030242. 16848617 PMC1518680

[91] Jeang K-T (2012). RNAi in the regulation of mammalian viral infections. BMC Biol. 10, 58. 10.1186/1741-7007-10-58. 22734679 PMC3383472

[92] Obbard DJ, Gordon KHJ, Buck AH, and Jiggins FM (2009). The evolution of RNAi as a defence against viruses and transposable elements. Philos. Trans. R. Soc. Lond. B Biol. Sci. 364, 99–115. 10.1098/rstb.2008.0168. 18926973 PMC2592633

[93] Cullen BR (2014). Viruses and RNA Interference: Issues and Controversies. J. Virol. 88, 12934–12936. 10.1128/JVI.01179-14. 25210170 PMC4249107

[94] Wilson RC, and Doudna JA (2013). Molecular Mechanisms of RNA Interference. Annu. Rev. Biophys. 42, 217–239. 10.1146/annurev-biophys-083012-130404. 23654304 PMC5895182

[95] Balgoma D, Gil-De-gómez L, and Montero O (2020). Lipidomics Issues on Human Positive ssRNA Virus Infection: An Update. Metabolites 10, 1–22. 10.3390/METABO10090356. PMC7569815 32878290

[96] Melendez-Villanueva MA, Trejo-Á vila LM, Galán-Huerta KA, and Rivas-Estilla AM (2021). Lipids fluctuations in mosquitoes upon arboviral infections. J. Vector Borne Dis. 58, 12–17. 10.4103/0972-9062.313961. 34818858

[97] Havranek KE, Ballista JMR, Hines KM, and Brindley MA (2021). Untargeted Lipidomics of Vesicular Stomatitis Virus-Infected Cells and Viral Particles. Viruses 14, 3. 10.3390/V14010003. 35062207 PMC8778780

[98] Petit T, Diderich JA, Kruckeberg AL, Gancedo C, and Van Dam K (2000). Hexokinase Regulates Kinetics of Glucose Transport and Expression of Genes Encoding Hexose Transporters in Saccharomyces cerevisiae. J. Bacteriol. 182, 6815–6818. 10.1128/JB.182.23.6815-6818.2000. 11073928 PMC111426

[99] Shen L, Hu P, Zhang Y, Ji Z, Shan X, Ni L, Ning N, Wang J, Tian H, Shui G, (2021). Serine metabolism antagonizes antiviral innate immunity by preventing ATP6V0d2-mediated YAP lysosomal degradation. Cell Metab. 33, 971–987.e6. 10.1016/j.cmet.2021.03.006. 33798471

[100] Meléndez-Hevia E, de Paz-Lugo P, and Sánchez G (2021). Glycine can prevent and fight virus invasiveness by reinforcing the extracellular matrix. J. Funct.Foods 76, 104318. 10.1016/j.jff.2020.104318.

[101] Escaffre O, Halliday H, Borisevich V, Casola A, and Rockx B (2015). Oxidative stress in Nipah virus-infected human small airway epithelial cells. J. Gen. Virol. 96, 2961–2970. 10.1099/JGV.0.000243/CITE/REFWORKS. 26297489 PMC4635479

[102] Jamaluddin M, Tian B, Boldogh I, Garofalo RP, and Brasier AR (2009). Respiratory Syncytial Virus Infection Induces a Reactive Oxygen Species-MSK1-Phospho-Ser-276 RelA Pathway Required for Cytokine Expression. J. Virol. 83, 10605–10615. 10.1128/JVI.01090-09. 19706715 PMC2753134

[103] Garofalo RP, Kolli D, and Casola A (2013). Respiratory Syncytial Virus Infection: Mechanisms of Redox Control and Novel Therapeutic Opportunities. Antioxid. Redox Signal. 18, 186–217. 10.1089/ars.2011.4307. 22799599 PMC3513983

[104] Sugai A, Sato H, Takayama I, Yoneda M, and Kai C (2017). Nipah and Hendra Virus Nucleoproteins Inhibit Nuclear Accumulation of Signal Transducer and Activator of Transcription 1 (STAT1) and STAT2 by Interfering with Their Complex Formation. J. Virol. 91, e01136–17. 10.1128/JVI.01136-17. 28835499 PMC5640859

[105] Koliopoulos MG, Lethier M, Van Der Veen AG, Haubrich K, Hennig J, Kowalinski E, Stevens RV, Martin SR, Reis E Sousa C, Cusack S, and Rittinger K (2018). Molecular mechanism of influenza A NS1mediated TRIM25 recognition and inhibition. Nat. Commun. 9, 1820–1913. 10.1038/s41467-018-04214-8. 29739942 PMC5940772

[106] Mitchell HD, Eisfeld AJ, Sims AC, McDermott JE, Matzke MM, Webb-Robertson B-JM, Tilton SC, Tchitchek N, Josset L, Li C, (2013). A network integration approach to predict conserved regulators related to pathogenicity of influenza and SARS-CoV respiratory viruses. PLoS One 8, e69374. 10.1371/journal.pone.0069374. 23935999 PMC3723910

[107] Tchitchek N, Eisfeld AJ, Tisoncik-Go J, Josset L, Gralinski LE, Bécavin C, Tilton SC, Webb-Robertson B-J, Ferris MT, Totura AL, (2013). Specific mutations in H5N1 mainly impact the magnitude and velocity of the host response in mice. BMC Syst. Biol. 7, 69. 10.1186/1752-0509-7-69. 23895213 PMC3750405

[108] Tisoncik-Go J, Gasper DJ, Kyle JE, Eisfeld AJ, Selinger C, Hatta M, Morrison J, Korth MJ, Zink EM, Kim Y-M, (2016). Integrated omics analysis of pathogenic host responses during pandemic H1N1 influenza virus infection: the crucial role of lipid metabolism. Cell Host Microbe 19, 254–266. 10.1016/j.chom.2016.01.002. 26867183 PMC5271177

[109] Sims AC, Mitchell HD, Gralinski LE, Kyle JE, Burnum-Johnson KE, Lam M, Fulcher ML, West A, Smith RD, Randell SH, (2021). Unfolded Protein Response Inhibition Reduces Middle East Respiratory Syndrome Coronavirus-Induced Acute Lung Injury. mBio 12, e0157221. 10.1128/mbio.01572-21. 34372702 PMC8406233

[110] Davidson AD, Williamson MK, Lewis S, Shoemark D, Carroll MW, Heesom KJ, Zambon M, Ellis J, Lewis PA, Hiscox JA, and Matthews DA (2020). Characterisation of the transcriptome and proteome of SARS-CoV-2 reveals a cell passage induced in-frame deletion of the furin-like cleavage site from the spike glycoprotein. Genome Med. 12, 68. 10.1186/S13073-020-00763-0/FIGURES/5. 32723359 PMC7386171

[111] Evans VC, Barker G, Heesom KJ, Fan J, Bessant C, and Matthews DA (2012). De novo derivation of proteomes from transcriptomes for transcript and protein identification. Nat. Methods 9, 1207–1211. 10.1038/nmeth.2227. 23142869 PMC3581816

[112] Silver JD, Ritchie ME, and Smyth GK (2009). Microarray background correction: maximum likelihood estimation for the normal-exponential convolution. Biostatistics 10, 352–363. 10.1093/biostatistics/kxn042. 19068485 PMC2648902

[113] Gentleman RC, Carey VJ, Bates DM, Bolstad B, Dettling M, Dudoit S, Ellis B, Gautier L, Ge Y, Gentry J, (2004). Bioconductor: open software development for computational biology and bioinformatics. Genome Biol. 5, R80. 10.1186/gb-2004-5-10-r80. 15461798 PMC545600

[114] Smyth GK (2005). Limma: linear models for microarray data. In Bioinformatics and Computational Biology Solutions Using R and Bioconductor (Springer), pp. 397–420.

[115] Norbeck AD, Monroe ME, Adkins JN, Anderson KK, Daly DS, and Smith RD (2005). The utility of accurate mass and LC elution time information in the analysis of complex proteomes. J. Am. Soc. Mass Spectrom. 16, 1239–1249. 10.1016/j.jasms.2005.05.009. 15979333 PMC1769320

[116] Nakayasu ES, Nicora CD, Sims AC, Burnum-Johnson KE, Kim Y-M, Kyle JE, Matzke MM, Shukla AK, Chu RK, Schepmoes AA, (2016). MPLEx: a Robust and Universal Protocol for Single-Sample Integrative Proteomic, Metabolomic, and Lipidomic Analyses. mSystems 1, e00043–16. 10.1128/mSystems.00043-16. 27822525 PMC5069757

[117] Zimmer JSD, Monroe ME, Qian W-J, and Smith RD (2006). Advances in Proteomics Data Analysis and Display Using an Accurate Mass and Time Tag Approach. Mass Spectrom. Rev. 25, 450–482. 10.1002/mas.20071. 16429408 PMC1829209

[118] Jaitly N, Monroe ME, Petyuk VA, Clauss TRW, Adkins JN, and Smith RD (2006). Robust algorithm for alignment of liquid chromatography-mass spectrometry analyses in an accurate mass and time tag data analysis pipeline. Anal. Chem. 78, 7397–7409. 10.1021/ac052197p. 17073405

[119] Monroe ME, Tolic N, Jaitly N, Shaw JL, Adkins JN, and Smith RD (2007). VIPER: an advanced software package to support high-throughput LC-MS peptide identification. Bioinforma. Oxf. Engl. 23, 2021–2023. 10.1093/bioinformatics/btm281. 17545182

[120] Kim Y-M, Nowack S, Olsen MT, Becraft ED, Wood JM, Thiel V, Klapper I, Kühl M, Fredrickson JK, Bryant DA, (2015). Diel metabolomics analysis of a hot spring chlorophototrophic microbial mat leads to new hypotheses of community member metabolisms. Front. Microbiol. 6, 209. 10.3389/fmicb.2015.00209. 25941514 PMC4400912

[121] Hiller K, Hangebrauk J, Jäger C, Spura J, Schreiber K, and Schomburg D (2009). MetaboliteDetector: Comprehensive Analysis Tool for Targeted and Nontargeted GC/MS Based Metabolome Analysis. Anal. Chem. 81, 3429–3439. 10.1021/ac802689c. 19358599

[122] Dautel SE, Kyle JE, Clair G, Sontag RL, Weitz KK, Shukla AK, Nguyen SN, Kim Y-M, Zink EM, Luders T, (2017). Lipidomics reveals dramatic lipid compositional changes in the maturing postnatal lung. Sci. Rep. 7, 40555. 10.1038/srep40555. 28145528 PMC5286405

[123] Aguilar HC, Aspericueta V, Robinson LR, Aanensen KE, and Lee B (2010). A quantitative and kinetic fusion protein-triggering assay can discern distinct steps in the nipah virus membrane fusion cascade. J. Virol. 84, 8033–8041. 10.1128/JVI.00469-10. 20519383 PMC2916531

[124] Zamora JLR, Ortega V, Johnston GP, Li J, André NM, Monreal IA, Contreras EM, Whittaker GR, and Aguilar HC (2020). Third Helical Domain of the Nipah Virus Fusion Glycoprotein Modulates both Early and Late Steps in the Membrane Fusion Cascade. J. Virol. 94, e00644–20. 10.1128/jvi.00644-20. 32669342 PMC7495395

[125] Zamora JLR, Ortega V, Johnston GP, Li J, and Aguilar HC (2021). Novel Roles of the N1 Loop and N4 Alpha-Helical Region of the Nipah Virus Fusion Glycoprotein in Modulating Early and Late Steps of the Membrane Fusion Cascade. J. Virol. 95, e01707–20. 10.1128/JVI.01707-20. 33568505 PMC8104108

[126] Matzke MM, Waters KM, Metz TO, Jacobs JM, Sims AC, Baric RS, Pounds JG, and Webb-Robertson B-JM (2011). Improved quality control processing of peptide-centric LC-MS proteomics data. Bioinforma. Oxf. Engl. 27, 2866–2872. 10.1093/bioinformatics/btr479. PMC3187650 21852304

[127] Webb-Robertson B-JM, Matzke MM, Jacobs JM, Pounds JG, and Waters KM (2011). A statistical selection strategy for normalization procedures in LC-MS proteomics experiments through dataset-dependent ranking of normalization scaling factors. Proteomics 11, 4736–4741. 10.1002/pmic.201100078. 22038874 PMC3517140

[128] Boyle EI, Weng S, Gollub J, Jin H, Botstein D, Cherry JM, and Sherlock G (2004). GO::TermFinder–open source software for accessing Gene Ontology information and finding significantly enriched Gene Ontology terms associated with a list of genes. Bioinformatics 20, 3710–3715. 10.1093/bioinformatics/bth456. 15297299 PMC3037731

[129] Heberle H, Meirelles GV, da Silva FR, Telles GP, and Minghim R (2015). InteractiVenn: a web-based tool for the analysis of sets through Venn diagrams. BMC Bioinf. 16, 169. 10.1186/s12859-015-0611-3. PMC4455604 25994840

[130] Babicki S, Arndt D, Marcu A, Liang Y, Grant JR, Maciejewski A, and Wishart DS (2016). Heatmapper: web-enabled heat mapping for all. Nucleic Acids Res. 44, W147–W153. 10.1093/nar/gkw419. 27190236 PMC4987948

[131] Croft D, O’Kelly G, Wu G, Haw R, Gillespie M, Matthews L, Caudy M, Garapati P, Gopinath G, Jassal B, (2011). Reactome: a database of reactions, pathways and biological processes. Nucleic Acids Res. 39, D691–D697. 10.1093/nar/gkq1018. 21067998 PMC3013646

[132] Webb-Robertson B-JM, McCue LA, Waters KM, Matzke MM, Jacobs JM, Metz TO, Varnum SM, and Pounds JG (2010). Combined Statistical Analyses of Peptide Intensities and Peptide Occurrences Improves Identification of Significant Peptides from MS-Based Proteomics Data. J. Proteome Res. 9, 5748–5756. 10.1021/pr1005247. 20831241 PMC2974810

[133] Webb-Robertson BJM, Matzke MM, Datta S, Payne SH, Kang J, Bramer LM, Nicora CD, Shukla AK, Metz TO, Rodland KD, (2014). Bayesian Proteoform Modeling Improves Protein Quantification of Global Proteomic Measurements. Mol. Cell. Proteomics 13, 3639–3646. 10.1074/MCP.M113.030932. 25433089 PMC4256511

[134] Aguilar HC, Matreyek KA, Filone CM, Hashimi ST, Levroney EL, Negrete OA, Bertolotti-Ciarlet A, Choi DY, McHardy I, Fulcher JA, (2006). N-Glycans on Nipah Virus Fusion Protein Protect against Neutralization but Reduce Membrane Fusion and Viral Entry. J. Virol. 80, 4878–4889. 10.1128/jvi.80.10.4878-4889.2006. 16641279 PMC1472062

[135] Schindelin J, Arganda-Carreras I, Frise E, Kaynig V, Longair M, Pietzsch T, Preibisch S, Rueden C, Saalfeld S, Schmid B, (2012). Fiji: an open-source platform for biological-image analysis. Nat. Methods 9, 676–682. 10.1038/nmeth.2019. 22743772 PMC3855844

[136] McDermott JE, Shankaran H, Eisfeld AJ, Belisle SE, Neuman G, Li C, McWeeney S, Sabourin C, Kawaoka Y, Katze MG, and Waters KM (2011). Conserved host response to highly pathogenic avian influenza virus infection in human cell culture, mouse and macaque model systems. BMC Syst Biol. 5, 190. 10.1186/1752-0509-5-190. 22074594 PMC3229612

[137] Li C, Bankhead A 3rd, Eisfeld AJ, Hatta Y, Jeng S,, Chang JH, Aicher LD, Proll S, Ellis AL, Law GL, Waters KM, Neumann G, Katze MG, McWeeney S, and Kawaoka Y (2011). Host regulatory network response to infection with highly pathogenic H5N1 avian influenza virus. J Virol. 85, 10955–10967. 10.1128/JVI.05792-11. 21865398 PMC3194976

