# Supplementary material for: Multi-platform omics analysis of Nipah virus infection reveals viral glycoprotein modulation of mitochondria
Source: Cell Rep. Author manuscript; Available in PMC 2025 May 23. (PMC12100452; doi:10.1016/j.celrep.2025.115411)
Supplement: 1 [file NIHMS2069123-supplement-1.pdf]

Cell Reports, Volume *44*

Supplemental information

Multi-platform omics analysis of Nipah virus infection reveals viral glycoprotein modulation of mitochondria

Gunner P. Johnston, Fikret Aydemir, Haewon Byun, Emmie de Wit, Kristie L. Oxford, Jennifer E. Kyle, Jason E. McDermott, Brooke L. Deatherage Kaiser, Cameron P. Casey, Karl K. Weitz, Heather M. Olson, Kelly G. Stratton, Natalie C. Heller, Viraj Upadhye, I. Abrrey Monreal, J. Lizbeth Reyes Zamora, Lei Wu, D.H. Goodall, David W. Buchholz, Joeva J. Barrow, Katrina M. Waters, Ruth N. Collins, Heinz Feldmann, Joshua N. Adkins, and Hector C. Aguilar

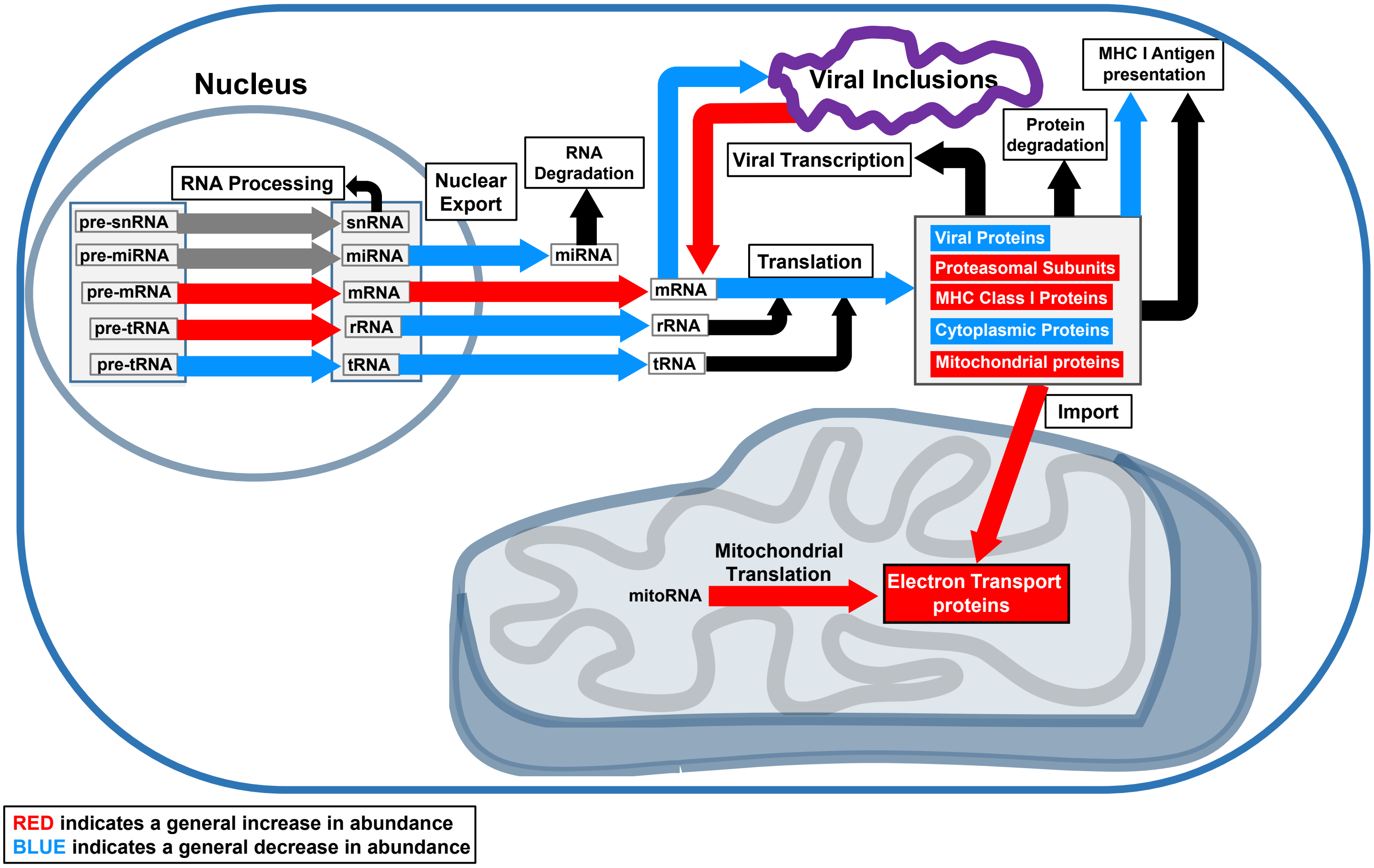

Figure S1. Overview of upregulated (red) and downregulated (blue) pathways using P value of <0.05, during viral infection, related to Figure 6.

				Pval (hpi)				Log2FC (hpi)				Trends (hpi)			
**REFERENCE**	**ACCESSION**	**DISPLAY NAME**	**# of PEPTIDES**	4	8	12	16	4	8	12	16	4	8	12	16
AAK50540.1	Q9IK92	NCAP_NIPAV	90		0	0	0	-0.92		7.39		0	2	2	2
AAK50541.1	Q9IK91	PHOSP_NIPAV	109	0.94	0.01	0	0	0.15	0.42	3.83	9.5	0	2	1	2
AAK50542.1	Q997F1	C_NIPAV	6			0	0					0	0	2	2
AAK50543.1	Q9IK90	MATRX_NIPAV	59		0.01	0.02	0	0.73	-3.91	2.71		0	2	2	2
AAK50544.1	Q9IH63	FUS_NIPAV	26	0.74		0	0	-1.33	1.99	2.25	7.2	0	0	2	1
AAK50545.1	Q9IH62	GLYCP_NIPAV	50		0	0	0	0.47		1.62	2.59	0	2	2	2
AAK50546.1	Q997F0	L_NIPAV	105			0	0	0.74		1.15	2	0	0	2	2
AAK50547.1	Q997F2	V_NIPAV	70		0	0	0	1.18		2.37	4.68	0	2	2	2

Table S1. Expression of Nipah virus proteins in a time-course, related to Figure 1. Detection of Nipah virus proteins indicating successful infection through proteomics analysis for 4, 8, 12 and 16 hpi.

Test performed	Classifier	Count.query	Count.universe	%.query	%.universe	p-value	FDR.q-value	Fold.change
8 hpi								
Positive log2FC								
Sub class	PE(	22-Nov	53/516	50	10.27	0	0	4.87
Specific chains by all	Glycerophospholipid with the chain 16:1	Jul-36	44/658	19.44	6.69	0.038	0.49	2.91
Negative log2FC								
Main class	PC	15-Oct	146/516	66.67	28.29	0.017	0.067	2.36
Sub class	PC(O-	15-Apr	22/516	26.67	4.26	0.029	0.144	6.25
Chain characteristics	Saturated	20/24	413/892	83.33	46.3	0.001	0.004	1.8
Specific chain	24:00:00	24-Mar	9/892	12.5	1.01	0.031	0.431	12.39
Total number of DB by all	PC with a total number of chain unsaturation of 0	10-Aug	51/146	80	34.93	0.04	0.101	2.29
12 hpi								
Positive log2FC								
no significant findings								
Negative log2FC								
Sub class	TG(	44/55	58/516	80	11.24	0	0	7.12
Chain characteristics	Monounsaturated	37/76	292/892	48.6842	32.74	0.011	0.085	1.49
Specific chain	18:01	24/76	122/892	31.5789	13.68	0	0.008	2.31
16 hpi								
Positive log2FC								
Category	Sphingolipid	13/51	61/516	25.49	11.82	0.026	0.04	2.16
Sub class	DG(	Aug-51	24/516	15.69	4.65	0.015	0.057	3.37
Sub class	GM3(d	Aug-51	15/516	15.69	2.91	0.002	0.028	5.4
Total chain carbon by all	Glycerophospholipid with a total number of chain carbon of 18	27-Jun	17/372	22.22	4.57	0.011	0.137	4.86
Negative log2FC								
Chain characteristics	Saturated	53/90	413/892	58.89	46.3	0.034	0.238	1.27
Specific chain	16:00	22/90	124/892	24.44	13.9	0.028	0.745	1.76
Total number of DB by all	Glycerophospholipid with a total number of unsaturation of 0	19/33	94/372	57.58	25.27	0.001	0.004	2.28
Specific chains by all	Glycerophospholipid with the chain 16:0	16/61	85/658	26.23	12.92	0.019	0.166	2.03
Specific chains by all	Glycerophospholipid with the chain 14:0	Sep-61	31/658	14.75	4.71	0.013	0.166	3.13
Specific chains by all	PC(with the chain 14:0	26-Jul	14/162	26.92	8.64	0.039	0.287	3.12
Enrichment test performed: EASE score test.								

Table S3. Lipid Enrichment Analysis, related to Figure 5. Lipid analysis highlighting the upregulated (positive log2FC) and downregulated (negative log2FC) lipids at 8, 12, and 16 hpi.

Found in NiV infection					
	F only	G only	F and G	Accession #	F and G co-transfection Uniprot Protein Names (Homo Sapiens)
Proteins found in infection	6	8	74		
Protein found in transfection	11	11	84		
% found in infection	55%	73%	88%		
list, in infection	HSPE1	ACADM	AARS2	Q5JTZ9	Alanine tRNA ligase, mitochondrial
	MRPL11	COX4I1	ACADM	P11310	Medium-chain specific acyl-CoA dehydrogenase, mitochondrial
	MRPL19	CYCS	ACADSB	P45954	Short/branched chain specific acyl-CoA dehydrogenase, mitochondrial
	NDUFV2	HADHA	ACAT1	P35610	Sterol O-acyltransferase 1
	PRDX3	IDH3B	ACO2	Q99798	Aconitate hydratase, mitochondrial
	SUCLG2	SHMT2	AIFM1	O95831	Apoptosis-inducing factor 1, mitochondrial
		TP53	ATP2A2	P16615	Sarcoplasmic/endoplasmic reticulum calcium ATPase 2
		UQCRB	ATP5A1	P25705	ATP synthase subunit alpha, mitochondrial
			ATP5B	P06576	ATP synthase subunit beta, mitochondrial
			ATP5D	P30049	ATP synthase subunit delta, mitochondrial
			ATP5O	P48047	ATP synthase subunit O, mitochondria
			C19orf70	Q5XKP0	MICOS complex subunit MIC13
			CHCHD3	Q9NX63	MICOS complex subunit MIC19
			CKMT1A	P12532	Creatine kinase U-type, mitochondrial
			CKMT1B	P12532	Creatine kinase U-type, mitochondrial
			CLPP	Q16740	ATP-dependent Clp protease proteolytic subunit, mitochondrial
			CLPX	O76031	ATP-dependent Clp protease ATP-binding subunit clpX-like, mitochondrial
			COX7A2	P14406	Cytochrome c oxidase subunit 7A2, mitochondrial
			CPOX	P36551	Oxygen-dependent coproporphyrinogen-III oxidase, mitochondrial
			DAP3	P51398	28S ribosomal protein S29, mitochondrial, MRP-S29, S29mt
			DES	P17661	Desmin
			DLD	A0A024R713	Dihydrolipoyl dehydrogenase
			ECH1	Q13011	Delta(3,5)-Delta(2,4)-dienoyl-CoA isomerase, mitochondrial
			ECHS1	P30084	Enoyl-CoA hydratase, mitochondrial
			ECSIT	Q9BQ95	Evolutionarily conserved signaling intermediate in Toll pathway, mitochondrial
			ETFA	P13804	Electron transfer flavoprotein subunit alpha, mitochondrial, Alpha-ETF
			FECH	P22830	Ferrochelatase, mitochondrial
			GFM1	Q96RP9	Elongation factor G, mitochondrial, EF-Gmt
			GLRX5	Q86SX6	Glutaredoxin-related protein 5, mitochondrial
			GLUD1	P00367	Glutamate dehydrogenase 1, mitochondrial
			GLUD2	P49448	Glutamate dehydrogenase 2, mitochondrial
			GOT2	P00505	Aspartate aminotransferase, mitochondrial
			GRPEL1	Q9HAV7	GrpE protein homolog 1, mitochondrial
			GRSF1	Q12849	G-rich sequence factor 1, GRSF-1
			HADH	Q99714	3-hydroxyacyl-CoA dehydrogenase type-2
			HADHA	P40939	Trifunctional enzyme subunit alpha, mitochondrial
			HSD17B10	Q99714	3-hydroxyacyl-CoA dehydrogenase type-2
			HSPA9	P38646	Stress-70 protein, mitochondrial
			HSPD1	P10809	60 kDa heat shock protein, mitochondrial
			IDH3A	P50213	Isocitrate dehydrogenase [NAD] subunit alpha, mitochondrial
			IMMT	Q16891	MICOS complex subunit MIC60
			LETM1	O95202	Mitochondrial proton/calcium exchanger protein
			LRPPRC	P42704	Leucine-rich PPR motif-containing protein, mitochondrial
			MDH2	P40926	Malate dehydrogenase, mitochondrial
			MIPEP	Q99797	Mitochondrial intermediate peptidase
			MRPL12	P52815	39S ribosomal protein L12, mitochondrial
			MRPL39	Q9NYK5	39S ribosomal protein L39, mitochondrial
			MRPL40	Q9NQ50	39S ribosomal protein L40, mitochondrial
			MRPS11	P82912	28S ribosomal protein S11, mitochondrial
			MRPS27	Q92552	28S ribosomal protein S27, mitochondrial
			MTHFD1L	Q6UB35	Monofunctional C1-tetrahydrofolate synthase, mitochondrial
			NDUFAF4	Q9P032	NADH dehydrogenase [ubiquinone] 1 alpha subcomplex assembly factor 4
			NDUFB10	O96000	NADH dehydrogenase [ubiquinone] 1 beta subcomplex subunit 10
			PDHA1	P08559	Pyruvate dehydrogenase E1 component subunit alpha, somatic form, mitochondrial
			PDHB	P11177	Pyruvate dehydrogenase E1 component subunit beta, mitochondrial
			PHB	P35232	Prohibitin
			PMPCA	Q10713	Mitochondrial-processing peptidase subunit alpha
			PMPCB	O75439	Mitochondrial-processing peptidase subunit beta
			PPIF	P30405	Peptidyl-prolyl cis-trans isomerase F, mitochondrial
			PTCD3	Q96EY7	Pentatricopeptide repeat domain-containing protein 3, mitochondrial
			SDHA	P31040	Succinate dehydrogenase [ubiquinone] flavoprotein subunit, mitochondrial
			SFXN1	Q9H9B4	Sideroflexin-1
			SHMT2	P34897	Serine hydroxymethyltransferase, mitochondrial
			SLC25A22	Q9H936	Mitochondrial glutamate carrier 1, GC-1
			STOML2	Q9UJZ1	Stomatin-like protein 2, mitochondrial
			TAMM41	Q96BW9	Phosphatidate cytidylyltransferase, mitochondrial
			TIMM44	O43615	Mitochondrial import inner membrane translocase subunit TIM44
			TOMM40	O96008	Mitochondrial import receptor subunit TOM40 homolog
			TOMM70A	O94826	Mitochondrial import receptor subunit TOM70
			TST	Q16762	Thiosulfate sulfurtransferase
			TUFM	P49411	Elongation factor Tu, mitochondrial
			UQCRC1	P31930	Cytochrome b-c1 complex subunit 1, mitochondrial
			VDAC1	P21796	Voltage-dependent anion-selective channel protein 1, VDAC-1, hVDAC1
			VIM	P08670	Vimentin

Table S5. Mitochondria sub-category of transfection proteomics data, related to Figure 6 and Figure 7. The upregulated proteins were compared to mock by statistical significance and peptide abundance ≥4. The items were then analyzed with Cytoscape to sub-categorize.

Name	Backbone	Tag
NiV N	pCAGGS	Myc
NiV P	pCAGGS	T7
NiV C	pCAGGS	E-tag
NiV V	pCAGGS	6xH
NiV W	pCAGGS	AU5
NiV M	pCMV	Flag
NiV F	pCDNA3.1	Flag
NiV G	pCDNA3.1	HA
NIV L	pCAGGS	no tag
RFP	pCDNA3.1	HA

Table S6. List of plasmids utilized in transfections, related to Figure 7. The codon optimized sequences for NiV genes were inserted into pCAGGs, pCDNA3.1, or pCMV-3 vector with tags. All plasmids were tested for expression using antibodies against protein tags in HEK293T cells.
